# 3D-Printed MEMS in Italy

**DOI:** 10.3390/mi15060678

**Published:** 2024-05-22

**Authors:** Matilde Aronne, Valentina Bertana, Francesco Schimmenti, Ignazio Roppolo, Annalisa Chiappone, Matteo Cocuzza, Simone Luigi Marasso, Luciano Scaltrito, Sergio Ferrero

**Affiliations:** 1 ChiLab Laboratory, Politecnico di Torino (PoliTo), Via Lungo Piazza d’Armi 6, 10034 Chivasso, Italy; matilde.aronne@polito.it (M.A.); matteo.cocuzza@polito.it (M.C.); simone.marasso@polito.it (S.L.M.); luciano.scaltrito@polito.it (L.S.); sergio.ferrero@polito.it (S.F.); 2 Department of Applied Science and Technology, Politecnico di Torino (PoliTo), Corso Duca Degli Abruzzi 24, 10129 Turin, Italy; francesco.schimmenti@polito.it; 3 Department of Chemistry, Biology and Biotechnology, University of Perugia, Via Elce di Sotto 8, 06123 Perugia, Italy; 4 Department of Chemical and Geological Science, University of Cagliari, Cittadella Universitaria Blocco D, S.S. 554 Bivio per Sestu, 09042 Monserrato, Italy; annalisa.chiappone@unica.it; 5 CNR-IMEM, Parco Area delle Scienze 37/A, 43124 Parma, Italy

**Keywords:** MEMS, 3D printing, additive manufacturing, NEMS, AM technologies, AM materials, MEMS sensors, MEMS actuator, microfluidic devices

## Abstract

MEMS devices are more and more commonly used as sensors, actuators, and microfluidic devices in different fields like electronics, opto-electronics, and biomedical engineering. Traditional fabrication technologies cannot meet the growing demand for device miniaturisation and fabrication time reduction, especially when customised devices are required. That is why additive manufacturing technologies are increasingly applied to MEMS. In this review, attention is focused on the Italian scenario in regard to 3D-printed MEMS, studying the techniques and materials used for their fabrication. To this aim, research has been conducted as follows: first, the commonly applied 3D-printing technologies for MEMS manufacturing have been illustrated, then some examples of 3D-printed MEMS have been reported. After that, the typical materials for these technologies have been presented, and finally, some examples of their application in MEMS fabrication have been described. In conclusion, the application of 3D-printing techniques, instead of traditional processes, is a growing trend in Italy, where some exciting and promising results have already been obtained, due to these new selected technologies and the new materials involved.

## 1. Introduction

Microelectromechanical systems (MEMS) combine electric functions with mechanical ones in a micrometric device [1]. Common examples of MEMS are sensors, like accelerometers or gyroscopes; actuators, like magnetic actuators; and microfluidic devices. Their manufacture has changed since their first appearance, with the use of polymers for MEMS fabrication, like polymethylmethacrylate (PMMA), polydimethylsiloxane (PDMS), or epoxy, instead of the traditionally used silicon. Not only have such constitutive materials been subjected to an evolution over the years, but fabrication technologies have also been affected by the advent of new ones. Traditional techniques, such as the wet bulk micromachining process or dry bulk micromachining process, have some disadvantages; the need for a prior photolithography process incurs high costs (due to requirements for specialised operators, costly procedures and materials, and dedicated infrastructure such as clean rooms) and a long fabrication time (for example, due to the preparation of masks) [2]. Moreover, the removal of material from the substrate region, which makes the silicon process a subtractive manufacturing process, limits the possible obtainable geometries [3]. These are some of the reasons which lead to the finding of alternative fabrication methods. Other solutions that have been introduced, such as the surface micromachining process, likewise have their drawbacks, because of the need for a sacrificial layer etching step, which imposes a drying step that can cause microstructure deformation and affect substrate structure adhesion. This behaviour is also linked to the slow liquid etchant evaporation and high surface tension at microscale [4].

Nowadays, the additive manufacturing (AM) world is the new frontier in the fabrication of MEMS devices, thanks to the latest advancements in 3D-printing technologies that allow one to reach the microscale. The additive manufacturing of metals and polymers is more and more used to produce parts or finite objects because of its advantages, like faster prototype fabrication, the easy modification and redesign of the product, less wasted material, and more geometrical complexity being allowed. Not all of the available technologies are suited for MEMS fabrication. Some 3D-printing processes offer a limited range of available materials, a bad resolution at microscale, a questionable accuracy and reproducibility, and post-processing procedures required to improve mechanical and surface finishing [5]. However, some AM technologies show a high resolution up to the nanometre range, together with high-quality surface finishing and parts’ geometry fabrication [6].

The 3D printing of MEMS devices is gaining ground, and many Italian research groups are focusing their attention on AM techniques for MEMS fabrication for their multiple advantages. Such 3D-printing technologies can help to avoid misalignment during the anisotropic etching process, reducing the problems of undesired under-etching; they facilitate the fabrication of 3D shapes that can be easily tailored, varying the process parameters [1]; they also ensure lower costs, together with the mass production of high-precision devices [7], that is a big advantage for the MEMS industry. Related to AM technologies for MEMS, there are also some challenges and issues that should be considered: the achievable features resolution relies on the chosen material and the printing technique, and for the same technique, the result can change when changing the printing parameters. Lastly, using polymeric or ceramic materials, it is necessary to face the linear and/or volumetric shrinkage of the printed part, due to polymerisation and a thermal post-processing step [1].

Nevertheless, the use of 3D-printing technologies is widely spread for the fabrication of micro- and nanoscale devices (MEMS and NEMS) in different emerging fields, such as biomedical engineering [8], soft robotics [9], optics and optoelectronics [10], printed sensors [11], and lab-on-a-chip [12]. In this review, we give an overview of the state of the art regarding the fabrication of MEMS through AM technology, focusing our attention on the panorama of Italian research on the topic. The review also aims to emphasise the variety and number of research projects in the Italian context related to the 3D printing of complex devices, following the growing trend towards additive manufacturing processes as key enabling technologies. Indeed, according to the Research and Markets Report on Additive Manufacturing [13], the global market size is estimated to reach USD 76.2 billion by 2030, with respect to a USD 16.8 billion turnover in 2022. The Italian government, aware of this trend, is actively supporting the development and adoption of additive manufacturing (AM) technologies through various initiatives. The last one was within the framework of ‘PNRR—Transizione 4.0’: with a fund of EUR 13.4 billion, the Ministry of Enterprises and Made in Italy is supporting the digital transition of companies focusing on Industry 4.0 technologies, including AM. It offers tax credits for investments in capital goods, intangible assets, R&D, innovation, and training related to AM. Moreover, the government supports the creation of AM competence centres, providing training, technical assistance, and access to AM equipment for businesses and researchers. An example is CIM4.0: born from the cooperation of the Politecnico and University of Turin, together with 22 partner businesses, it provides services specialising in AM, and it offers strategic and operational support, helping companies to innovate and keep their processes highly competitive. There also exist other bodies which are created as cultural associations and which promote the knowledge diffusion of AM. One of the best known is AITA (Associazione Italiana Tecnologie Additive), which today boasts almost ten years of activity.

The 3D printing of MEMS has been investigated, starting from the analysis of different 3D-printing techniques suitable for MEMS and the materials used for MEMS device fabrication, and finally reporting recent outcomes and updates. Hence, Section 2 is dedicated to a theoretical introduction to AM technologies for MEMS printing, with a description of their working principles and the main elements that are required for fabricating 3D parts. Section 3 gives an overview of the specific application of the previously described technologies in the fabrication of MEMS sensors, MEMS actuators, and microfluidic devices, focusing on the Italian context. Section 4 explains the materials used for AM technologies, and it also reports examples of their application in the 3D printing of MEMS devices. Section 5 summarises all the previously discussed topics and provides some closing remarks.

## 2. AM Technologies Classification

The International Organisation of Standardisation (ISO) has classified AM technology into seven groups based on the printing process (ISO 52900:2021): vat photopolymerisation, material extrusion, material jetting, binder jetting, powder bed fusion, sheet lamination, and direct energy deposition [14]. Below, there is a list of the commonly used ones in the MEMS sector, which are vat photopolymerisation, material extrusion, material jetting, and powder bed fusion. To the best of our knowledge, no examples of MEMS printing have been reported using binder jetting, direct energy deposition and sheet lamination technologies. As regards binder jetting, in which a glueing agent is deposited as ink on a powder bed, this is probably due to material compatibility with the typical MEMS working environment. Concerning direct energy deposition, the high number of parameters to control, and the need for coupling this technology with a CNC machining phase for the printed parts, probably make this technique quite complex to be used for MEMS. In the case of sheet lamination, the achievable printing accuracy could be the reason why such a technology is not employed for printing micrometric and nanometric parts.

### 2.1. Vat Photopolymerisation

**Figure 1:**
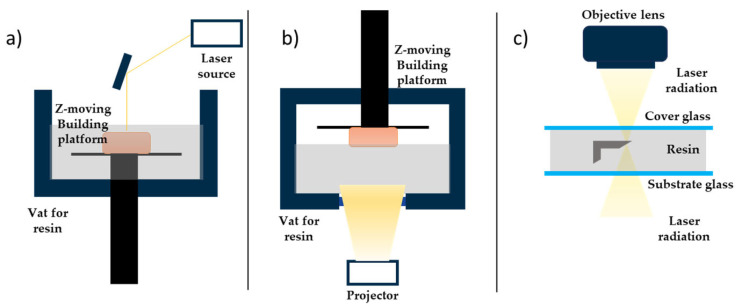
Working principle of vat photopolymerisation technologies: (**a**) stereolithography, (**b**) digital light projection, (**c**) two-photon polymerisation. The system components for each technology are listed.

Vat photopolymerisation is a broad term; it refers to different technologies for fabricating finite objects starting from a liquid photocurable resin, which is photopolymerised layer by layer using a laser or a projector. These systems can ensure the highest resolution and accuracy level, a good interlayer bonding (adhesion between consequent slices), and smooth surface, upon choosing the correct set of printing parameters. It is also possible to print more pieces at the same time, depending on the built plate dimensions. The printing process is fast enough, even if, for complex geometries and for bigger parts, it can take hours [5]. The technologies that are referred to as vat photopolymerisation are stereolithography, digital light projection, and two-photon polymerisation. A schematic representation of how these technologies work is provided in Figure 1.

#### 2.1.1. Stereolithography

The stereolithography (SL) printing process involves a liquid-filled vat, in which a plate can move along the Z axis, and an ultraviolet (UV) laser beam as input, in a galvanometric head that allows the beam to move around in the XY plane. For each layer growing, the plate goes down a one-layer thickness into the resin; then, the beam turns on and point-to-point photopolymerises the desired geometry. Some printers also have an after-recoating phase, during which a blade removes the excess resin before the photopolymerisation step, so they require less material to work [15]. The spot diameter in this technology is around tens of microns, which guarantees a better accuracy and control for the smaller features, especially for micrometric devices. SL-printed parts need to be post-processed: they should be cleaned for unreacted resin, commonly using isopropyl alcohol (IPA) or ethanol (EtOH), and they also have to be post-cured using a UV lamp, to polymerise the trapped unreacted groups and enhance their mechanical properties [5]. As for other technologies, support structures are required for all the pieces with overhangs, because their weight can cause their collapse.

#### 2.1.2. Digital Light Projection

The digital light projection (DLP) process involves a liquid-filled transparent vat, a plate that can move along the Z axis, in and out the vat, and a LED projector, that allows the irradiation of the XY plane with the layer geometry. For each layer, the plate goes down a one-layer thickness into the resin; then, the projector turns on and photopolymerises the layer following the desired geometry. After the printing process, a post-process step is required: the printed parts have to be cleaned for unreacted material, commonly using isopropyl alcohol (IPA) or ethanol (EtOH), and they also have to be post-cured using a UV lamp, to polymerise the unreacted groups and enhance their mechanical properties [5]. DLP parts have a good accuracy and resolution, smoother surface, lower cost, and faster fabrication, but they tend to have higher shrinkage-related problems.

#### 2.1.3. Two-Photon Polymerisation

Two-photon polymerisation (TPP, or 2PP) is an additive manufacturing technique that has features in common with other vat photopolymerisation techniques, like the use of lasers, but it does not require a liquid-filled vat. In TPP, light-sensitive material, the photoresist, is positioned on a glass substrate if it is a high-viscosity material, while it is positioned between two thin glass covers separated by spacers if it is a low-viscosity material [16]. The photopolymerisation of the photoresist occurs in the small volume of a drop that is selectively irradiated by a femtosecond laser beam at a near-IR wavelength, whose focal point defines the position of the polymerised voxel (three-dimensional pixel). The voxel dimensions are typically into the submicron range; that is why this technique allows the fabrication of submicrometric structures with a higher resolution, compared to single-photon lithography, and why it also allows the fabrication of complex 3D geometries without supports or multistep processes, because the printed parts grow inside a photoresist drop [17]. There are some advantages in the use of TPP: firstly, it requires low light intensities, thanks to the two-photon absorption mechanism; secondly, it is possible to integrate the structure in different materials, changing the resin inside the droplet and dipping the lens inside it, if the structure is particularly tall; then, it ensures a structuring process that is more flexible and precise, assigning for each layer the special coordinates that the laser has to reach [16,17]. Some drawbacks are the slower printing processes that increase the printing time; difficulties in mass-scale production, because of the high printing time and the inability to print multiple objects together; the high printing resolution strongly depends on the numerical aperture of the objectives and, also, on the material’s interaction with the light during the process. This dependence of the printing outcomes on the material composition is due to the intensity threshold model. A window with two limits is defined, within which the polymerisation can occur; the lower limit identifies the lowest possible light intensity that starts photopolymerisation, while the upper one identifies the light intensity that starts to burn the material or cause bubble formation [16].

### 2.2. Material Extrusion

Material extrusion AM techniques rely on solid-state materials, mainly thermoplastic ones, that can be extruded from a nozzle when heated. These technologies have a simple setup, which is usually cheaper than the ones for vat photopolymerisation technologies, and a lower energy demand; these elements make it one of the most widespread AM technologies, even at the industrial level [18].

#### Fused Filament Fabrication

**Figure 2:**
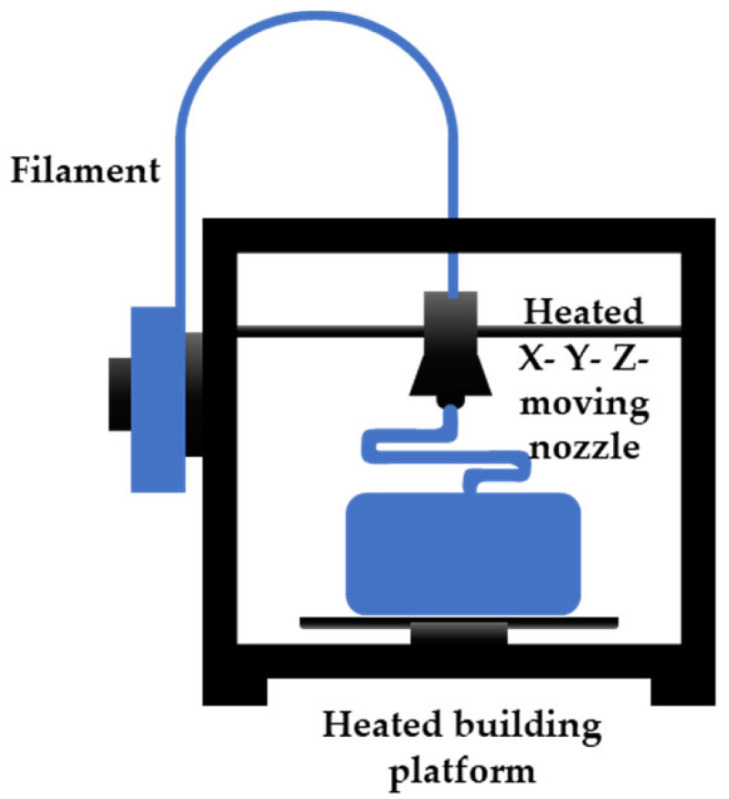
FFF working principle, with system components listed.

Fused filament fabrication (FFF) technology belongs to the extrusion-based AM technologies, because it involves the extrusion of a filament through a nozzle that heats the material above its melting point. The semi-molten filament comes from a spool, that continuously feeds the printing head and that can contain a thermoplastic or a metal material. The nozzle can move along three axes, to construct geometries layer by layer on the heated printing bed, ensuring the better adhesion of the printing objects. A schematic representation of the technology’s working principles is shown in Figure 2. Some FFF printers use a second heated head to simultaneously print a second material that can be used as sacrificial material, for supports, or as reinforcing material, for some applications [5,19]. On one hand, this printing technique has different advantages, like a low cost, the high performance of the printers, and the wide variety of materials that can be used, even transparent and biocompatible ones. Moreover, FFF printers are user friendly and environmentally friendly, and the chemical or UV post-curing of the printed parts is not a required step [5,20]. On the other hand, some disadvantages can be identified, such as a low resolution and low surface quality, due to the filament thickness, bad surface finishing, and a long printing time for complex objects [5].

### 2.3. Material Jetting

Material jetting (MJ) AM technology is defined by the ASTM as a technique which involves the deposition of feedstock material droplets in a selective way [21]. This printing process relies on the deposition of photosensible materials, drop by drop, on a build plate, and their consecutive photopolymerisation with a lamp, whose wavelength is between 190 nm and 400 nm. The used materials are stocked into an air-excluding tank, then delivered to the printing head through a transmission line. In the printing head, the ink is heated up to enhance its flowability through the nozzles, from which is jetted. The printing head can be also equipped with a roller, acting like a doctor blade, that ensures the deposition of a thin layer. Finally, there is a lamp, flashing light to induce photopolymerisation. The head can move along the X axis, while the build plate can move along the Z axis; the deposition along the Y axis is determined by the placement of the printing head nozzles, and they are activated during the printing phase, according to the geometry. As for other AM techniques, MJ requires support structures for overhangs, so a gel-like sacrificial material is needed, and then, it has to be removed at the end of the process, mechanically or chemically. This class of AM technologies has different pros: it ensures a thin layer thickness, so staircase effects can be avoided, and thin wall features can be printed with a low surface roughness and high resolution; there is a good finishing of the final parts; undesirable effects of draughts or dirt are prevented, thanks to the use of a closed printing chamber; and there is an easy detachment of the printed parts from the printing bed [21]. The commonly used MJ technology is multi-jet modelling, also called PolyJet technology by some manufacturers.

#### 2.3.1. Multi-Jet Modelling/PolyJet

Multi-jet modelling (MJM) technology uses material-jetting techniques, but the printing head can deposit two materials at the same time; typically, the first one is the building material, while the second one is the sacrificial material [21]. The printing process proceeds layer by layer, as usual, but it offers a better level of detail, with a resolution of 656 × 656 × 1600 DPI (XYZ), and a minimum layer thickness of 16 µm [12], with an accuracy of 0.1 mm [22]. The printed parts have to be post-processed, in order to remove the support material and to prepare the parts for their applications, for example, for use as mould for PDMS. This technology has different advantages, like a high accuracy and resolution at a millimetric scale, a good surface quality, and the use of different materials depending on the application; however, it has some drawbacks, like a limited range of materials, a low build process, and poor performances at microscale or with high-aspect ratio objects [23].

#### 2.3.2. Inkjet Printing

**Figure 3:**
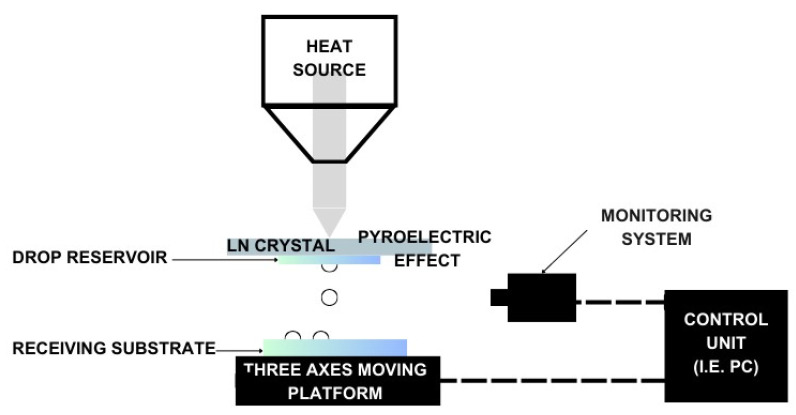
Pyroelectric–Electrohydrodynamic (pyro-EHD) system components and working phase.

The inkjet printing (IJP) technique is a material-jetting process that is part of the non-contact direct-writing class of printing technologies. The inks, that are a solution or dispersion of nanoparticles into a solvent, are deposited on a flexible or rigid substrate by a nozzle, filled with material through the hydraulic system that connects the ink reservoir and the printing head. The printing head is itself a MEMS actuator, and it can be a thermal, a piezoelectrical, or an electrodynamical actuator. The deposition system can eject material with a continuous flow (called a continuous IJP) or drop by drop (called a drop-on-demand delivery system) along the Z axis, mainly. Both the printing techniques can process geometries with a resolution of 20 µm or higher, by adjusting the applied voltage, the printing bed speed, the chemical composition of the ink, and its viscosity and surface tension. The printing bed moves in the XY plane, following a vector-based path from a CAD drawing, and it can be heated, to promote solvent fast evaporation [16]. An evolution of inkjet printing technology is the Pyroelectric–Electrohydrodynamic (pyro-EHD) system, a nozzle-free inkjet technology that includes a heating system, three-axes moving platform, and a monitoring system. The ink reservoir is a microscope glass slide, placed under a Lithium Niobate (LN) crystal positioned on the platform. The platform motion control system has a high linear precision to ensure a maximum travel speed of 30 mm/s, and it deposits the ink on a printing bed, made of a cover glass, that can host the substrate. The material is ejected after the crystal heats up, because of the breaking of liquid surface tension by a strong field gradient that generates instability. This pyroelectric effect is a temperature-dependent effect, as shown by Equation (1):

(1) $$\Delta P_{i}=p_{i}\Delta T$$

where p_i_ is the pyroelectric coefficient (−4 × 10^−5^$\frac{C}{m^{2}K}$), and it takes a high electrical field, such as 10^6^–10^8^ V/cm, due to high uncompensated charges density. This phenomenon induces liquid suspension to generate micro-drops that are deformed by the electrical field, and then they are deposited on the substrate. The jetting phase is monitored by the monitoring system, which is composed of a fast camera, an optical zoom lens, and a blue LED light (Figure 3).

This IJP technique is slowly spreading into the MEMS fabrication world because of its high resolution and the possibility of printing 3D structures directly onto existing devices with a fine spatial accuracy, even if there is a big issue about reproducibility, due to the distance between the substrate and the reservoir, which needs to be actively controlled during the printing process [24].

### 2.4. Powder Bed Fusion

**Figure 4:**
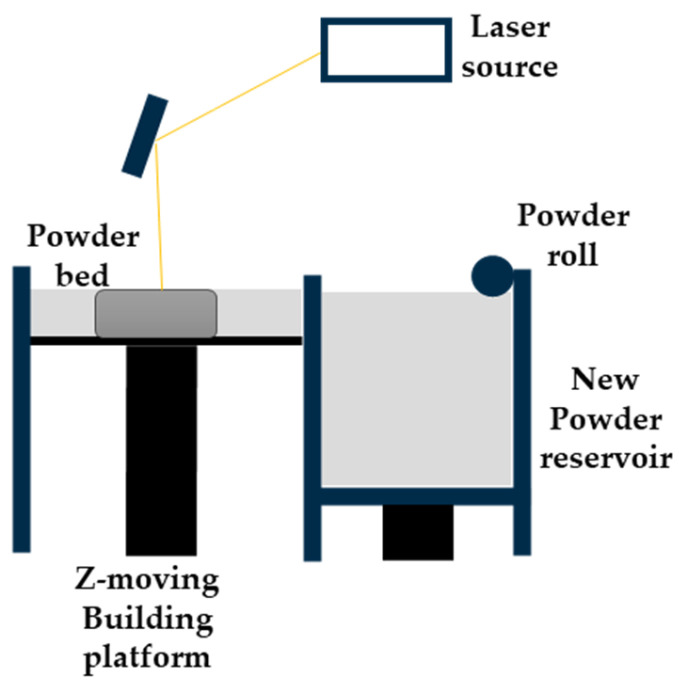
Powder bed fusion technology working principles and system components.

Powder bed fusion (PBF) processes are AM technologies where a thermal energy source selectively fuses a powder, which can be metallic or polymeric. A fixed amount of the powder is spread onto the building bed using a recoater, usually a roller, to obtain a smooth and homogeneous layer that guarantees the density and the homogeneity of the layer. After that, the thermal source is activated, and the fusion of the powder particles starts in the selected regions. Commonly, the energy source is a laser beam that is focused on different points of the area in the XY plane. Other possible energy sources are high-power electron beams or infrared light, which heats up regions where a heat-conducting liquid has been previously dispersed. Then, the consolidation of the single layer happens, because of the coalescence between particles due to the temperature increasing in the area. The heated part solidifies with the cooling down of the powder bed; lastly, the printing bed is lowered a one-layer thickness, to deposit a new layer of powder [16]. One of the widespread powder bed fusion techniques is selective laser sintering, for polymeric materials, and selective laser melting, for metals. The technology’s working principles and components are shown in Figure 4.

#### 2.4.1. Micro Metal Laser Sintering

Micro metal laser sintering (µMLS) is a powder bed fusion AM technology that works with metals, for high-resolution and high-surface quality parts. The working principle of this technique is the same as for all powder bed fusion processes, with a building bed for metallic powders, which are spread using a recoater. The spread powder is composed of sub-micrometric particles, which are sintered using a laser system, typically a neodymium:YAG (Nd:YAG) fibre laser for higher absorptivity and quality, which operates in a continuous or pulsed mode, in the range 100 W–2 kW, with a spot size between 50 nm and 180 nm [25,26]. Together with the laser head, a mirror-based system, made of a galvanoscanner and focusing lenses, helps the positioning of the laser spot at high speeds, ensuring high accuracy [26]. The achievable layer thickness with this technique is ~1 µm; that is why the particles used should have nanometric dimensions, preventing powder agglomeration during printing the process [25]. For the alignment of the layers, a metrology setup is employed for the nano-positioning system, with a resolution of 200 nm for the coating gap control and with a resolution of 10 nm for approaching the optical station for each laser scanning [25,26]. The printing substrate is fixed on the Z axis using a vacuum chuck, which has a heater, to lower the thermal gradient between the printing part and the powder but also to reduce the process energy needing. The whole printing system is positioned on a vibration isolation stage, to avoid possible damage to the parts [25]. To avoid an undesired chemical reaction due to gases in the printing close chamber, an inertial gas flow, like nitrogen or argon flow, is applied [26]. At the end of the printing process, the final object has to be cleaned of unsintered powder, using ultrasonication, and it has to be post-processed to enhance its mechanical or electrical characteristics [25]. The post-processing phase involves, first, a washing step to remove unsintered parts, then an annealing step at high temperature in an oven for a certain time. µMLS has several advantages, like the possibility of manufacturing movable parts and assemblies at microscale with a high resolution; the vertical stacking of the printing parts can be accomplished, paying attention to the parts’ weight, without supports being needed, because the powder itself acts as support for overhangs and the stacked elements. The printed objects show a smooth surface and a high detail resolution and accuracy, even within complex geometries and shapes [27].

#### 2.4.2. Selective Laser Melting

Selective laser melting (SLM) is another 3D-printing technique that belongs to the family of PBF technologies, and it involves the melting and fusion of selected powder areas. As with other PBF processes, it starts with the deposition of a thin layer of powder, typically a metal one, on the printing bed inside the chamber. After that, the thermal source, a high-energy laser, is turned on and it selectively melts the material, according to the geometry, and, at the end, another powder layer is deposited above the melted and solidified one. During the process, the chamber can be filled with inert gases, like nitrogen or argon, to avoid oxidation, and it can also be pre-heated, to reduce the required energy for material melting. The commonly used laser system is the Nd:YAG fibre laser, at 1.06 µm, even if there is a trend to replace it with ytterbium and the YAG (Yb:YAG) fibre laser nowadays; because it is highly absorbed by metallic powders in the infrared region, it has a longer lifetime, and it has a lower thermal loading per unit power. When the printing process ends, the printed object is cleaned of non-fused powder excess, and it is removed from the platform manually or through other processes, like electrical discharge machining (EDM). As with other AM technologies, SLM has some limitations in the resolution and accuracy that can be achieved for the printed parts, and these are also linked to the printing parameters. The typical range of values for layer thickness is from 20 µm to 100 µm; the selection of the value is related to the particles’ size and their flowability, since bigger particles reduce the resolution and affect the build tolerance, while smaller ones tend to agglomerate and reduce the flowability, i.e., the deposition phase. Laser power, its scanning speed, and hatch spacing, are process parameters to consider in the optimisation phase of the printing process, because they determine if a single area is totally melted or if neighbouring areas are involved, and if the actual layer is fused with the previous one. Another important parameter is powder absorbance at the fixed wavelength, because it determines the volumetric energy density required for the process. It is strongly linked to the material—in particular, to its heat capacity and latent heat—and it is proportional to the mass of the material that has to be melted. If the energy dose is insufficient, the two subsequent layers cannot adhere, while if the laser energy is too high and the scanning speed is low, the high evaporation of the material can happen. If the hatch spacing is low, regular porosity can appear in the final part, due to the lack of fusion between two adjacent lines. However, after an optimisation step of the printing parameters, SLM can produce a complete, dense, near-net-shaped part, with a one-step process, without the need of intense post-processing treatments and without the need for binders, improving the printed object quality, the time for the process, and reproducibility of the printing process [28].

All the mentioned technologies have been grouped and classified in Table 1, summarising their working principles and their advantages and disadvantages.

## 3. AM Technology for MEMS Fabrication

In the Italian research scenario, various AM technologies have already been applied to the fabrication of MEMS, and here we report an overview of the devices presented in the published works, divided according to the employed fabrication technologies.

### 3.1. Vat Photopolymerisation

#### 3.1.1. Stereolithography

Many of the 3D-printed MEMS have been fabricated through SL technology, which is one of the most accurate and high-resolution techniques. One interesting application has been reported by Scaccabarozzi et al. [29]; they fabricated a holder for a quartz crystal microbalance for space applications. Their work relied on the SL printer FormLabs 3D printer, with a printing resolution of 0.05 mm, and on FormLabs resin (FLHTAM02), which ensures the right mechanical and thermal characteristics, to obtain a compact and lightweight solution. Their holder design was determined using a finite-element (FE) approach, referring to some requirements for the same devices for space applications. It presented two disks that had to be assembled to handle the crystal but ensuring the insertion of electrodes at the same time; there were also holes for screws, to preload the crystal. Comparison of FE simulations with the device’s mechanical characterisations underlined the agreement between the experimental behaviour and expectations, even if further experiments in a space-like environment were required to validate the applicability of the device as a space microbalance.

Other MEMS sensors fabricated through SL technology are the Coriolis mass flowmeter, by Pagani et al. [30], and the three-axes accelerometer by Zega et al. [31]. Both of these works report a double-step fabrication that involved SL 3D printing and copper wet metallisation, for a small size and a cheap and large-scale process. The first appearance of this two-step sensor’s fabrication was in 2018, when Zega et al. [32] made the first *Z*-axis accelerometer. They introduced the SL and wet metallisation of MEMS devices by studying them on three different single-ended configurations, which were first simulated using COMSOL^®^ Multiphysics (https://www.comsol.com/comsol-multiphysics, accessed on 10 April 2024). In the first configuration, two elongated suspending (300 µm out-of-plane thickness) springs allowed the motions of a 2 mm-thick proof mass, while in the second configuration, two folded suspending springs were fabricated, reducing the footprint by a 1.42 factor. Last, in the third configuration, two diagonal folded springs were introduced.

The simulated devices were fabricated and tested, showing good agreement between the simulation results and the experimental ones, a sign of the reliability of the design and fabrication flow. The first step of fabrication was the SL of the geometries, using DL260^®^ as a resin (digital wax systems (DWS), Thiene, Italy), which was loaded with 20%wt. of amorphous silica, and the DWS028J+ (DWS, Thiene, Italy) as the printer, equipped with a monochromatic actinic laser (Solid State BluEdgeBE-1500A/BE-1500AHR). The resin mechanical characteristics (2.2 GPa Young’s modulus, a 29.6 MPa tensile strength, and an elongation at the break of 1.7%) gave to the printed part the desired mechanical behaviour, while the printer characteristics (30 mW emitting power at 405 nm, a laser spot of 11 µm, and a lateral and vertical resolution of 10 µm) allowed the reaching of the required dimensions for the final part, like a 20 µm layer thickness. For the capacitive readout, the printed MEMS sensor required an electrically conductive surface, both on the suspended mass and on the electrodes, so they introduced the Cu wet metallisation of the devices. Firstly, the resin surface was pre-treated with an alkaline solution because it was dielectric, in order to activate the electroless CU deposition. Thanks to this step, a 0.5 µm Cu layer was obtained. That layer acted as a starting point for the Cu electrolytic deposition, so a second Cu layer of 1.5 µm layer was obtained. Lastly, the Cu-metallised structures were fixed to two Cu planar electrodes, with a Polyethylene Terephthalate (PET) sheet of 100 µm interposed for electrical insulation. These devices showed comparable or even better performances compared to state-of-the-art MEMS accelerometers, in terms of sensitivity, linearity, and noise, ensuring the full customisation of them at the same time [32].

In [30], rectangular spiral channels were fabricated following the already described fabrication process, ensuring a completely customisable and fully 3D-printed sensor, lowering costs, and maintaining the desired performance. This flowmeter’s overall dimensions were 60 mm in length and 47 mm in width, with a channel diameter of 1 mm (with 500 µm-thick walls). The so-fabricated flowmeter was mounted on a printed circuit board (PCB), connected to the electrodes for actuation and for the readout step [30]. The already-cited work [31] explains the fabrication of a 3D-printed three-axes accelerometer, made using the same process described above [32], the SL printing of the basic structure, and its wet metallisation. The innovative 3D design of this MEMS sensor is exploited for 3D symmetric acceleration measurements, and it included a suspended proof mass linked to the frames through folded springs. The designed geometry involved overall dimensions of 2.24 cm × 2.24 cm × 2.24 cm and a spring cross-section of 0.7 mm × 0.5 mm and a nominal gap between the mass and the fixed elements of 200 µm. This was possible thanks to the SL-printing technology, which overcame the limitations of traditional planar fabrication processes, and it also allowed the fabrication of small features, around a few hundred µm, with higher-sensitivity performances, compared to state-of-the-art MEMS accelerometers. The 3D geometry of the sensor was determined using the Multiphysics simulation, to avoid spurious modes due to the springs in the operation frequency range. The final part was Cu wet metallised using two different approaches, to determine the best solution for the external metallic coverage. Then, the device was fixed on a PCB using a conductive glue for the electrical connection, and the electrodes were fixed to the three axes using 200 µm PET sheets as a dielectric interlayer. Despite the manual assembly of these devices, these 3D-printed MEMS devices showed good performances, and, in addition, this third example displayed a symmetry of quality factor and low value of thermal noise [31]. The double-step fabrication process described was also applied in the fabrication of a MEMS actuator, like in [33], where a magnetic-actuated cantilever was made. This device, whose dimensions are 9 mm length, 0.6 mm width and 0.2 mm thickness, was printed like the sensors reported above, and then it was Cu metallised to obtain a first conductive layer. After that, a permalloy of NiFe layer was electrodeposited, and then it was covered by an electrodeposited Co layer. These two soft magnetic alloys presented a lack of permanent magnetisation that made them suitable for cantilever functioning, avoiding the interference of a permanent magnetic field and ensuring the correct response of the device to the external trigger. The movement of the cantilever was activated using a magnet, and the relative distance between them was varied and the deflection measured. The behaviour of this device seemed to be pseudo-linear at low magnetic fields; that made it controllable in actuation and suitable for some critical applications, like microfluidic control or electrical switching [33].

Sensors and actuators have not been the only MEMS devices fabricated via SL technology; the first steps were already taken in this direction for microfluidic devices in the early 2000s. Microfluidic chips are devices that contain a certain number of microchannels, whose diameters range from 1 to hundreds of µm. They can be made of different materials, like silicon, glass, and polymers, through several fabrication processes, like lithographic techniques. These microchannel collections are used as liquid and gas transport paths for target operations and analysis, improving their performances thanks to miniaturisation. Miniaturised device dimensions imply a reduction in the required working fluid volume, less waste, a more efficient analysis, alaminar flow that permits the observation of diffusion phenomena, more efficient heat dissipation [34], and an increase in the surface–volume ratio.

In recent years, the use of SL technology has expanded for lab-on-a-chip and Bio-MEMS device fabrication, due to its potentialities and performance. SL 3D printing allows the freedom of shape and design flexibility of the final device, a higher aspect ratio compared to traditional silicon-derived fabrication techniques, the availability of biocompatible and transparent materials for microfluidic chip fabrication, and a simplified fabrication chain, which also implies lower costs and a higher production volume and fabrication speed. It is also true that some critical issues are still open for this kind of microfluidic device production technology, in particular, the compliance with strict dimensional tolerances and the desired mechanical properties, which remain stable over time [35]. These critical points have been faced by different works that show the possible application of SL technology for microfluidic device fabrication; here, we report some of them.

**Figure 5:**
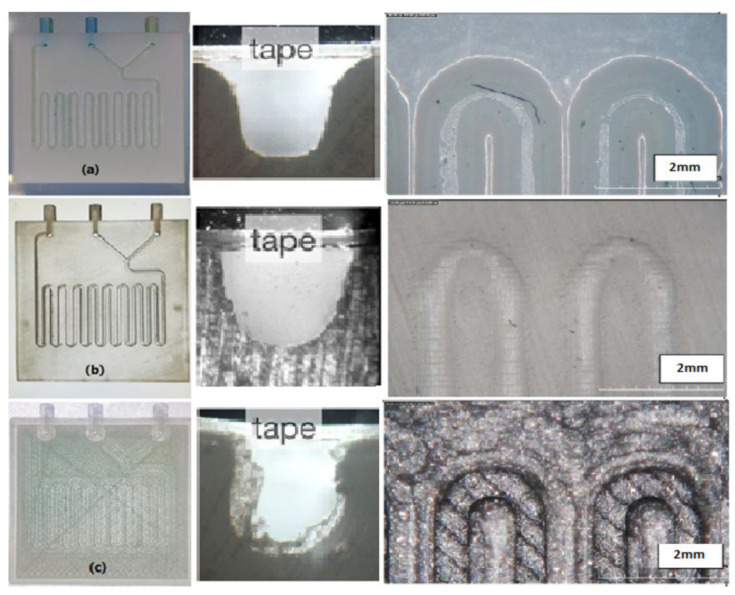
Fabricated micromixers by three different methods of printing. (**a**) Micromixer with PolyJet in cross-section and top view, (**b**) micromixer with SLA in cross-section and top view, (**c**) micromixer with FDM in cross-section and top view. Reprinted from [36].

Zeraatkar et al. [36] investigated the performances of different 3D-printing technologies for microfluidic device fabrication, comparing three of these techniques: SL, FFF, and PolyJet (Figure 5).

For instance, they fabricated micromixers, which are microfluidic components that help fluid dilution for chemical or biological reactions, relying on diffusion and chaotic advection phenomena, that can be passively or actively started. These mixing mechanisms require complex elements, like ridges and grooves, to ensure a correct mixing of the fluids, because the laminar flow alone takes a long time to mix the fluids, allowing only diffusion between them [37].

The design chosen in the work of Zeraatkar et al. [36] presented two inlets for the two fluids that were connected to the serpentine, which was a square-like channel consisting of 18 turns, whose dimensions were 600 µm wide and 600 µm high, and, at the end, there was an outlet. The SL apparatus used was Formlabs Form 2, while the printed material was Clear Form V2 UV, to obtain a transparent object, whose channels were closed with a tape. The inner surface of the SL-printed device was smooth, the channel dimensions showed little differences from the nominal ones, and the device ensured a good mixing efficiency at different flow rates [36].

Another example of SL 3D printing for microfluidic device fabrication is represented by the work of Dallari et al. [10], whose aim was to combine microfluidic technology with an optical sensor for surface-enhanced Raman scattering (SERS) analysis. SERS is a powerful optical detection technique that relies on the localised surface plasmons (LSPs) of metallic nanostructures, which interact with an electromagnetic field scattered by the analysed samples, even at low sample concentration. The metallic nanostructures had to be placed inside the microfluidic chamber, where the fluid could interact with the metal nanostructures, generating a higher SERS signal after a mixing period. Microfluidic chips were made through the replica moulding of a mould 3D-printed using the XFAB 2500 HD stereolithography apparatus (DWS, Thiene, Italy), which uses a monochromatic actinic laser source, with a power of 30 mW at λ = 405 nm and a 50 µm laser spot diameter. The chosen resin is Vitra 430^®^ (from DWS, DWS, Thiene, Italy), and the printed geometry was replicated using PDMS. The printed mould dimensions were 25 mm × 35 mm, with channel dimensions of 100 µm × 100 µm that connected the three chambers (whose diameter was 3 mm). Inside the microfluidic channels, gold nanostars were immobilised, using an -NH_2_ group induced on the PDMS surface through silanisation with aminosilane. To seal the channels, the replicas were bonded to a 200 µm-thick layer of flat PDMS, via plasma activation of the surface. The coupling between the microfluidic devices and optical sensors guaranteed a high versatility to the analysis platform, tailorable device design, and tuneable properties, varying the nanostructure inside the geometry [10].

**Figure 6:**
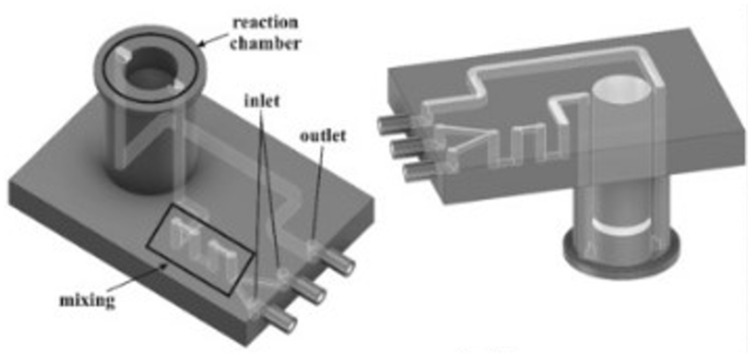
Silicon photomultiplier (SiPM) for ATP detection through bioluminescence. All the microfluidic features of the device are reported. Reprinted from Biosens Bioelectron, vol. 99, M. F. Santangelo, S. Libertino, A. P. F. Turner, D. Filippini, and W. C. Mak, “Integrating printed microfluidics with silicon photomultipliers for miniaturised and highly sensitive ATP bioluminescence detection”, pp. 464–470, January 2018, with permission from Elsevier [38].

Following the idea of reagent consumption reduction in optoelectronics devices for detection, Santangelo et al. [38] presented an integrated platform for ATP bioluminescence detection, coupling a microfluidic chip with silicon photomultipliers (SiPMs) to create a bioluminescent reader, reported in Figure 6. A SiPM is a solid-state light detector, made of a pixels array connected to a single common resistor that collects the output current level, where each pixel contains a single photon avalanche diode operating in Geiger mode, and a series of quenching resistors, to turn off the avalanche of the diode. This photodetector ensures a high quantum efficiency, high gain and speed, low operating voltage, and single-photon sensitivity. The use of microfluidic devices as a reagent-channelling and reaction chamber allowed working with low reagent concentrations and volume consumption, a low-cost solution that managed to detect small amounts of ATP. The microfluidic chip was fabricated using an SL printer (Form 1+, from Formlabs, Somerville, MA, USA) and a proprietary transparent resin (Clear Type 02, from Formlabs), and the open side of the channels and reaction chamber were sealed with regular adhesive tape. The chosen solution ensured cost-effectiveness, an easy and short design time, and a simple fabrication process, even if the geometry was quite complex. The chosen design involved two inlets, with an internal radius of 500 µm, connected to a series of serpentine mixers that ended in a channel, whose other end was connected to the reaction chamber. After the fluids mixture passed through the chamber, there was another channel that ended with the outlet port. The SiPM was placed in contact with the reaction chamber, and it comprised 25 pixels fabricated on the same silicon wafer portion, insulated one from the other optically and electrically with excavations filled by oxide or metal. The SiPM was inserted into a box, with a small hole for contacting the reagents region, and the whole system was contained by a dark box, to avoid optical noise. Compared to commercially available bioluminescence readers, this configuration showed a similar sensitivity and low noise, enabling the evaluation of small concentrations of ATP with a compact and low-cost solution that had good stability, reproducibility, and acceptable experimental errors. It opened up the fabrication of bioluminescence measurement systems for the real-time monitoring of biological events, for clinical diagnostics, and other applications in chemical and biological analysis [38].

#### 3.1.2. Digital Light Projection

There are some examples in the literature that report the use of DLP as a fabrication process for MEMS devices, thanks to its attractive features, like the fabrication of complex-shaped designs, a single-step process, lower costs, versatility, the tunability of the final part’s properties, changing materials and printing parameters, a high resolution, and high surface quality.

The possibility to integrate mechanical features, electrodes, and electronics for physical stimuli and for chemical or biological analysis has pushed the research to find the best method for lab-on-a-chip (LoC) fabrication, starting with microfluidic devices.

**Figure 7:**
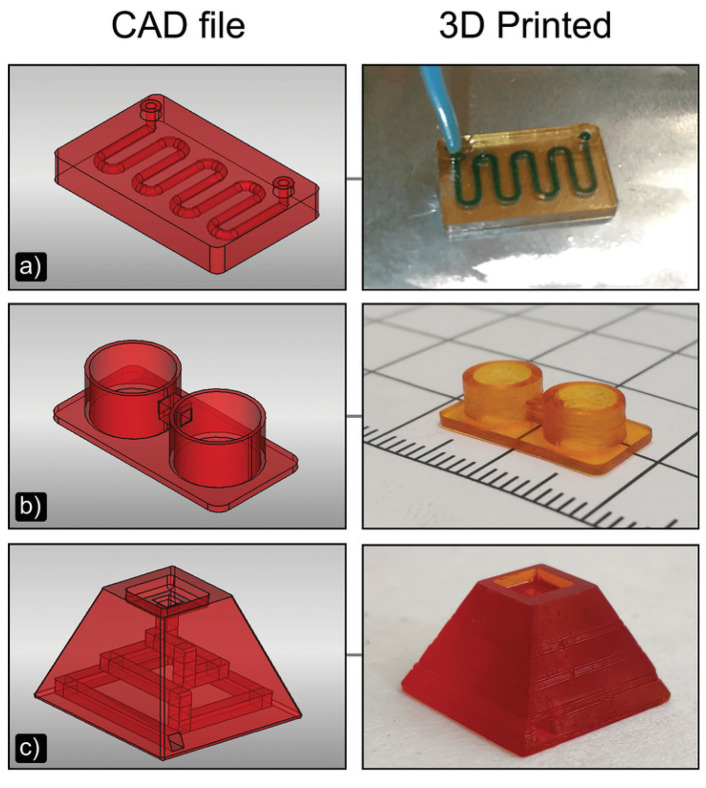
DLP-printed microfluidic objects compared to their CAD models. (**a**) 3D printed microfluidic chip with an s-shaped channel of 800 µm of diameter; (**b**) Two 3D printed wells connected by a 1 × 1 mm^2^ square section channel; (**c**) Trapezoidal 3D printed microfluidic chip with a 1 × 1 mm^2^ channel square section. Reprinted from Advanced Material Technology, vol. 5, no. 9, G. Gonzalez, A. Chiappone, K. Dietliker, C. F. Pirri, and I. Roppolo, “Fabrication and Functionalisation of 3D Printed Polydimethylsiloxane-Based Microfluidic Devices Obtained through Digital Light Processing”, September 2020, with permission from John Wiley and Sons [39].

Herein is reported, as a first example of DLP application to MEMS devices, in particular for microfluidic platform fabrication, the work of Gonzalez et al. [39]. In this study, they prepared a PDMS-like resin for microfluidic devices, and they used as a fabrication technique DLP 3D printing. Their resin contained an acrylate polydimethylsiloxane copolymer as the oligomer, TEGORad2800 (Evonik, Essen, Germany), a blend of phenyl bis(2,4,6-trimethylbenzoyl) phosphine oxide (BAPO), composed by a BAPO-methyl ester, a BAPO-isooctyl ester, and isooctanol as the photo-initiator, dispersed red 1 methacrylate (DR1-MA) as a visible-light absorber dye, and methyl methacrylate (MMA) monomer for solvating the dye. The microfluidic chip was fabricated using an Asiga PICO 2 DLP-3D printer (Asiga, Alexandria, Australia), which presents a LED light source at 405 nm, an XY resolution of 50 µm, and a Z resolution of 1 µm. The selected printing parameters were a layer thickness of 50 µm, light intensity of 20 mWcm^−2^, and exposure time of 10 s per layer. Different complex microfluidic platforms were printed, as reported in Figure 7, and the unreacted double bonds were used for attaching functional molecules on the channel surface, to enhance the surface properties compared to classical PDMS devices obtained through soft lithography. The printed samples showed high chemical stability, good mechanical properties, flexibility, stretchability, and high optical transparency, and their surface properties were proven to be easily and selectively modifiable; all these considerations demonstrated the compatibility of this fabrication process with the requirements for microfluidic platforms for several applications.

A further example of DLP’s application to microfluidic chips is the work of Bucciarelli et al. [40], which reported the optimisation process for device fabrication through 3D printing of a commercial transparent resin, GR-10 from pro3dure medical GmbH, that is also biocompatible, according to ISO-10993 [41]. This resin, made of bisphenol A-ethoxylate dimethacrylate (2 EO/Phenol) and BAPO, was 3D printed with an Asiga MAX X27 UV, from Asiga, whose characteristics are a printable volume of 51.8 mm × 29.2 mm × 75 mm, an XY resolution of 27 µm, and a high-power LED at 385 nm. The printed object had high-aspect ratio-critical features, namely, a series of squared pillars with 100 µm width and 1.2 mm height, inside the 2 mm-thick fluidic chamber. In order to compare the fluidic performances of traditional PDMS microfluidic chips obtained through replica moulding with the 3D-printed devices, they sealed the printed chip with a PDMS layer through oxygen plasma treatment, then they performed a fluidic test, inserting a mix of food dye and water using a syringe pump, for one week. The fabricated devices demonstrated that, by tuning the printing parameters, it was possible to achieve small features (down to 50 µm) with a high aspect ratio, up to 60, using DLP for the direct fabrication of microfluidic devices, while it was more difficult with replica moulding, because of the incomplete PDMS curing at the mould–PDMS interface, the material density and the detachment required force, due to surface roughness that should be smoothed with a post-treatment. Directly 3D-printed chips also showed a higher resolution, no leakage, high transparency, and a higher Young’s modulus and yield stress, even if the printed parts should be sealed with an additional step, and they also showed an excess in the XY dimension (around 28%) in respect to the nominal CAD dimension, probably linked to the higher exposure time required for high-aspect ratio structure fabrication. However, the intermediate step of channel sealing allowed the achievement of a deep cleaning of the piece, and the possibility to combine other fabrication technologies such as bioprinting or to insert elements like electrodes and sensors before the sealing [40].

Another application of DLP for MEMS device fabrication is represented by the work of Stassi et al. [42], which developed a micro-cantilever for biosensing. The developed device was 8 mm in length and 9 mm in width as a footprint area, and each cantilever had a width of 0.7 mm and a height of 0.2 mm. They chose DLP as the fabrication technology to overcome limitations in the production of resonant mechanical structures for mass sensing, such as the reaching of a high resolution, very low thickness, the need for functionalisation, and a long preparation time, because of the 3D-printing advantages described above and the use of polymers instead silicon as a surface material. The printing device was made of a home-made resin, whose composition included bisphenol A ethoxylate diacrylate (BEDA) as a monomer, acrylic acid (AA), as a functionalisation agent, bis-(2,4,6-trimethylbenzoyl)- phenyl phosphine oxide (Irgacure 819, BASF) as a photo-initiator, and reactive orange 16 (RO) as a dye to control light penetration during printing. This resin was printed with the Freeform Pico Plus 39 DLP printer from Asiga, (Alexandria, Australia) whose characteristics are XY resolutions of 39 µm, a layer thickness range from 10 to 100 µm, a build area of 50 mm × 30 mm × 150 mm, and a light-emitting diode light source that emits at 405 nm, with an intensity of 22 mW∙m^−2^; the selected printing parameters were a layer thickness of 25 µm and an exposure time of 0.8 s per layer. Thanks to the resin composition, they were able to control the number of available active groups on the surface of the cantilever, which helped in the enzymatic functionalisation of the device for biosensing application, while the use of 3D-printing technology reduced the time for fabrication and allowed the production of an array of a micro-cantilever for parallel measurement processes [42].

Finally, a fabrication technique that is projection-based is projection micro-stereolithography (PµSL) patented by the Boston Micro Fabrication (BMF) company (Maynard, MA, USA). An application example can be found in the work of Saitta et al. [43], which printed a micro-optofluidic device. PµSL is a special type of DLP 3D printing, which enables the rapid fabrication of complex 3D structures at microscale, layer by layer, thanks to the combination of traditional SL features with advanced digital technology for projection lithography [34]. This process has many advantages, like a short fabrication time, a high precision at microscale, and the possibility of printing complex shapes with a one-step process. The device fabricated in [43] showed optical and microfluidic features, because it had one channel along the Y axis for the optical fibres and a T-junction along X axis for fluid flow. This configuration was chosen for the detection of slug passages inside the device, reading the optical signals within the fibres. The fluids, with different refractive indexes, were inserted into the two inlets of the T-junction, and they flowed throughout the microfluidic channel. During their passage, they interacted with a light beam emitted from the optical fibre, transmitting it and scattering it according to their own refractive index. The other optical fibre detected the transmitted light, and the recorded signal was used for slug passage detection. The chosen material was HTL resin from BMF, because of its temperature stability that makes the final device suitable for repeated autoclave cycles, and also for its water contact angle, ensuring that no flow instability is generated into the channel, even if the resin’s optical properties are not optimal, compared to the PDMS that is traditionally used for microfluidic devices; the printer used for the device fabrication was the microArch^®^S140 ultra-high-resolution (10 µm) 3D printer, from BMF [43].

#### 3.1.3. Two-Photon Polymerisation

As previously stated, TPP technology allows the fabrication of geometries at a sub-nanometric range that are impossible to obtain with different alternatives. The group of Cojoc et al. [44] fabricated micro-optics with different shapes on top of optical fibres through TPP, because it ensures freedom for complexity, good accuracy and resolution, a few-fabrication-steps process, and the use of a transparent substrate and photosensitive materials with good optical properties. The first fabricated optic was a convergent optic with a curvature radius of 7 µm, the second one was an axicon lens with an apex angle of 117°, and the third one was ring-shaped, with a thickness of 0.9 µm. They used a custom-made setup for TPP, with a 100 fs pulse width, 80 MHz Ti: Sapphire laser oscillator as an excitation source, and they worked at 720 nm as a wavelength and 7.5 mW power. The facility also presented a fibre holder mounted on a platform and a piezo stage that could move along the X, Y, and Z axes with a maximum displacement of 80 µm. As a photo resin, they selected the UV-curing adhesive NOA 63 (Norland, Jamesburg, NJ, USA), that has an optimal sensitivity at 350–400 nm, a good adhesion to the glass substrates, a low cost, and a suitable reflective index. A drop of resin was positioned in the centre of a glass substrate, a microscope coverslip, and it was kept in close contact with the optical fibre end; the distance between the coverslip and the fibre end should be below 200 µm. The fabrication of 3D structures was achieved through the scanning of the sample, with two different strategies, which were annular scanning with a fixed constant step in the Z direction and annular scanning with a variable step in the Z direction, according to the desired shape. The optics-printing tests showed that different shapes of micro-structures could be obtained, with good optical performances and a fine control of complex features, making TPP a good alternative to traditional fabrication processes [44].

The second example of the TPP fabrication process for MEMS devices is present in the work of Stassi et al. [17]; they made rigid nano-resonators with a high quality factor using a TPP approach. They prepared their own photopolymeric liquid ink based on metal salts and photocurable groups; in particular, they chose metal chloride salts to dissolve into propylene glycol and an AA solution. The ink was crosslinked with TPP for the nanometric resolution required for the NEMS resonator, and the final printed part faced a densification step to eliminate the organic part and to consolidate the metal precursor, obtaining a rigid ceramic structure with a high Young’s modulus and low damping at the end. For that purpose, the metal precursors were neodymium (Nd) and YAG, because the Nd: YAG composite shows a high elastic modulus and intrinsic properties like a gain medium. They printed three different designs as resonators—clamped–clamped beams (bridges), single-clamped beams (cantilevers), and circular membranes—with a length range from 20 µm to 50 µm, a width range from 2 µm to 5 µm, and a thickness range between 250 nm and 2000 nm, using the Photonic Professional GT printer, from Nanoscribe GmbH (Eggenstein-Leopoldshafen, Germany). The printed objects showed high quality factors, up to 150,000, that depended on the device’s thickness; the thinner devices had a higher Q and high sensitivity, comparable to silicon-based NEMS, but the fabrication process was easier and faster, because it requires fewer fabrication steps and lower costs [17].

**Figure 8:**
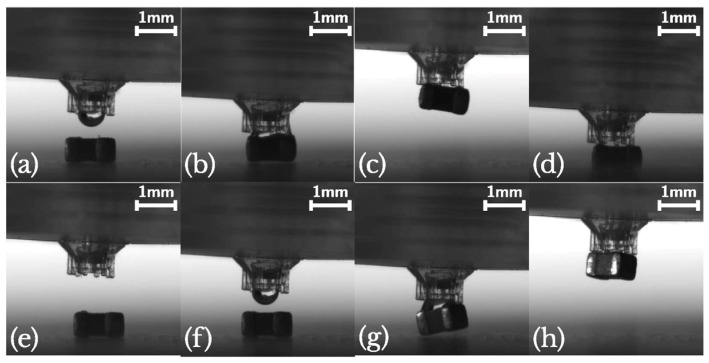
Microgripper working phases: component-gripping frames are (**a**–**c**) and (**g**,**h**); component-releasing frames are (**d**–**f**). Reprinted from [45].

Another interesting application of TPP technology in the MEMS world is represented by the work of Dehaeck et al. [45], which fabricated microgrippers that relied on capillary force to grip the component. The presence of a liquid layer helps the components to maintain a scratch-free surface, and it also excludes the influence of surface irregularities on the grip force. For this purpose, they developed a hybrid fabrication process, made of two steps, to obtain a final device with features at different-length scales—the centimetric and micrometric scale. The two fabrication processes were as follows: SL for the macroscopic part, the tip holder, that also comprised the opening for the connection of the tubes; TPP for the microgripper, which presented four pillars and a central hole for the water that was the liquid chosen for the capillary grip of the components. The selected TPP machine was Nanoscribe Photonics Professional GT, and the resist was Nanoscribe IP-L 780 (both from Nanoscribe GmbH, Eggenstein-Leopoldshafen, Germany), in a dip-in configuration using a 25 X objective. For a good adhesion and to avoid a mechanical/thermal mismatch of the two printed elements, they chose SL resin Autodesk PR48 and, as the SL machine, that Autodesk Ember 3D printer (both from Autodesk, San Francisco, CA, USA) that works with a UV projector with a pixel-size of 50 µm and a slicing distance of 25 µm. The microgripper dimensions were designed starting from the 1005 SMD capacitor as the component to grip; so, the rectangular-shaped design had a 0.5 mm × 0.5 mm surface, with a 0.5 mm overhang of the component’s surface compared to the gripper’s, that took the SMD so as to be picked up obliquely and to be released when the more exposed extremity met the surface, as shown in Figure 8.

The major challenge in this scenario was the positioning of the microscopic component on top of the macroscopic component, which required the direct manufacturing of the microgripper on the SL-printed device, after a pre-printing alignment step. First, the SL-printed part was positioned in the TPP printer, then the interface between the solid part and the liquid resist was found, to have a Z-reference for the print. Last, the position of the centre of the liquid channel was defined manually, to identify where the central hole should be fabricated. Thanks to this hybrid fabrication approach, they made microgrippers with a high resolution and highly complex design, but some issues were still unresolved. Sometimes, they found liquid leaks in the system, probably due to the limits of the manual alignment or cracks in the holder. They also noticed a micro-explosion during polymerisation with the Nanoscribe, perhaps linked to some debris floating in the IP-L resist. Last, the printing time was high, around 5 h, and the process was difficult to speed up in a cost-effective manner [45].

### 3.2. Material Extrusion

#### Fused Filament Fabrication

Material extrusion technology—in particular, FFF—is gaining ground in the MEMS scenario, for its low cost, its quite wide range of materials, its versatility, and the potential lightness of parts that is a more and more general requirement in several fields, like the automotive and avionic. Inertial MEMS sensors, as accelerometers and gyroscopes, are widely applied in these sectors, because they can measure the earth rate and estimate the heading angle without the need for other sensors, thus proving to be a necessary and essential tool for navigation. However, they present some drawbacks, like a high cost and high sensitivity to environmental conditions, especially the smaller ones, affecting measurements and reducing their applicability. A commonly adopted solution in inertial measurement units (IMUs) MEMS relies on a redundant configuration that can include redundant geometrical forms in the same IMUs, or the integration of multiple IMUs on the same system configuration, identifying and isolating failures, mitigating errors in the measurements, and increasing accuracy and robustness.

An example of MEMS redundant IMUs is presented by de Alteriis et al. [19], which proposed a packed navigation system composed of multiple IMUs mounted on a 3D-printed structure, to ensure protection to the sensors and to achieve a reduction in the weight and dimensions of the final part, with enhanced mechanical properties. In their work, they used the “Mark Two”, from Markforged™ (Waltham, MA, USA), as a 3D printer and an FFF machine with two extrusion head to deposit two materials in parallel; one was the base material, ONYX (from Markforged™), and one was the reinforcing material, carbon fibres. They printed a 5 cm × 5 cm × 5 cm cubic structure, composed of six panels; each of them was made up of 16 layers, whose thickness was 0.125 mm. The central eight layers were reinforced with carbon fibres, with a stacking sequence of a 0°, 45°, 90°, and 135° inclination, to obtain a higher mechanical resistance and performance even at higher temperatures, thanks to their controlled alignment and positioning, while the first and last four layers were made of ONYX alone. They evaluated some noise parameters, like bias instability and random walk, through Allan variance, and the internal temperature’s influence on these measures, to understand the performance and reliability of the system. The results showed that this 3D-printed IMUs system had the ideal shape factor for an Unmanned Aerial System, thanks to the customised design and high mechanical performances, due to the reinforcement with carbon fibres, and the accuracy and reliability of the measurements are comparable to a standard system [19].

To reduce thermal influence in IMU-MEMS accelerometers, the group of Ruzza et al. [46] evaluated their thermal behaviour on board, in a tilted configuration, mounting one of them into a homemade thermal chamber. This was composed of three parts: a thermoelectric cooling and heating element (TEC), a power driver, and temperature sensors; it was coupled with the tilting device, that allowed the performance of measurements during the inclination phase. The tilting device was made of a biaxial 3D-printed tilting frame and a couple of servomotors to control the inclination, and it was printed with an LAB54 printer, from 3DPRN, in ABS, at 210 °C with a velocity of 30 mm*s^−1^. The designed structure allowed a biaxial tilting of the chamber, in order to test the thermal response of accelerometers actioned on two axes, with low-cost, open-source, reproducible and easy-to -ecover components [46].

Another example of an inertial MEMS sensor fabricated with FFF is the work of Barile et al. [47], which presented a differential capacitance-based accelerometer, with a symmetrical structure, for an equal response along the three axes. They chose this fabrication technology because it allows one to speed up the fabrication process; it is easier compared to traditional one; it allows one to couple different materials during the same process in a simple way, assigning the correct material to each part of the object; and it also allows one to act quickly in the case of defects or malfunctions, substituting the faulty part rapidly. They fabricated three suspended proof masses using a Protopasta Conductive filament and a blend of polylactic acid (PLA) and carbon black, while the insulating structure, and the springs that suspend the masses were printed using only PLA; the supports were made of polyvinyl alcohol (PVA), and they were removed through water solubilisation after the printing process. They used as printer the E3D Tool Changer, from E3D, which had three different tools for each material, avoiding mixing and contamination. They tested the printed MEMS through a pulse response experiment, frequency response experiment, and input–output (I/O) characteristic evaluation, to obtain a complete sensor characterisation. They verified that the lower the mass density, the higher the resonance frequency, and the response linearity fell as the mass sensor decreased, while the slope of the I/O characteristic increased when the proof mass increased, so it had high sensitivity. These results indicated that it is possible to obtain, through FFF, a MEMS capacitive accelerometer, with a tailorable shape, performance, and material that can be varied in correlation to the application [47].

Moving from inertial MEMS sensors to microfluidic MEMS ones, a first example is the work of Moscato et al. [11], which presented a substrate-integrated waveguide (SIW) for the real-time characterisation of fluids under Ultra-Wideband (UWB). They printed two microstrip transmissions lines (the first one, 45 mm length, and the second one, 60 mm length; both had a width of 3 mm) which were fabricated on a t-glass substrate, whose thickness was 1.2 mm. They decided to adopt FFF technology to fabricate the microfluidic device because it allows the of manufacture of pipes and cavities directly on the substrate without the need for additional steps or silicon technology, obtaining a multi-folded pipe, with a square cavity, embedded into the dielectric substrate. The material used for 3D printing with Metal Plus, a machine from Printrbot that presents a vertical resolution of 0.1 mm suitable for small empty cavities, was t-glass, from Taulman3D, which is a PET derivative, with a low loss tangent and moderately high dielectric constant. The implemented design had two vertical holes for the inlet and the outlet, for continuous flow, and they had the same pipe diameter. The length of the pipes, their distance, and number were designed to optimise the filling of the SIW surface, together with the consideration of the limits of the manufacturing technology. Thanks to this fabrication technique, they were able to print a sensor with a high quality factor, high sensitivity, and high accuracy, as their tests showed, paving the way for future application with organic fluids [11].

On the same page is the work of Di Giampaolo and Di Natale [48], who fabricated a microwave sensor through the FFF printing of PLA. Their study stems from the need for a highly sensitive, reliable, and accurate sensor for biochemical analysis, that allows working with a small volume of samples, so they designed a very thin and flat T-resonator, that used the resonant frequency and quality factor to measure material properties with some modification, such as introducing a stub line (0.5 mm diameter) in the substrate, to perturb the resonator behaviour, which showed the different resonant frequency of the material at specific points, allowing one to distinguish the material inside the channel. Their 0.8 mm-thick substrate also had a honeycomb structure, to reduce undesired losses and to increase the electric field in the channel. The fabricated part was tested to evaluate its performance, and they found that this structure was able to work with only 12 µL of fluid, it was highly sensitive to permittivity variation varying the working liquid, and it also permitted the integration of more channels to increase the efficiency of the analysis, obtaining a high-throughput platform for a microfluidic assay [48].

The group of Zeraatkar et al. [37] have presented another application for a microfluidic device printed through FFF, a Y-shaped micromixer with ridges inside the channel. They selected crystal-clear PLA, from Fabbrix (Ruvo di Puglia, Italy), as the material, and the Ultimaker S5 printer, from Ultimaker (Utrecht, The Netherlands), as the FFF machine, using two different nozzles: the 0.25 mm-diameter nozzle, for an infill width of 200 µm, and the 0.4 mm-diameter nozzle, for an infill width of 600 µm. They chose a layer height of 100 µm, an infill line distance of 200 µm and 600 µm and an infill extrusion angle of 60°, a printing temperature of 200 °C, a built plate temperature of 60 °C, and a printing speed of 70 mm*s^−1^. The printed geometry included two inlets and one outlet, whose diameters were 0.5 mm and 0.75 mm respectively; two inlet channels of 600 µm in length and width; and one mixing channel 52 mm long, whose width was 900 µm and length was 600 µm. The ridges inside the channel were a consequence of the printing parameters; in particular, they came from the width of the infill filament, the layer thickness, infill orientation, and distance between adjacent filaments; so, they measured the final ridges’ dimensions after the printing process, to define the correlation between the printing parameters and ridges formation. They also correlated them with the mixing performances of the printed devices, measured using, as fluids, methylene blue dissolved in distilled water and distilled water flows, and they found that devices with a channel width of 600 µm and channel height of 300 µm had a ridges height–depth ratio of 0.33 and they also showed the best mixing properties, ensuring a short time and requiring a relatively short channel length. Lastly, these features were fabricated without the need for complex surface-patterning machining or external devices for active mixing, reducing the complexity and cost of the whole system.

**Figure 9:**
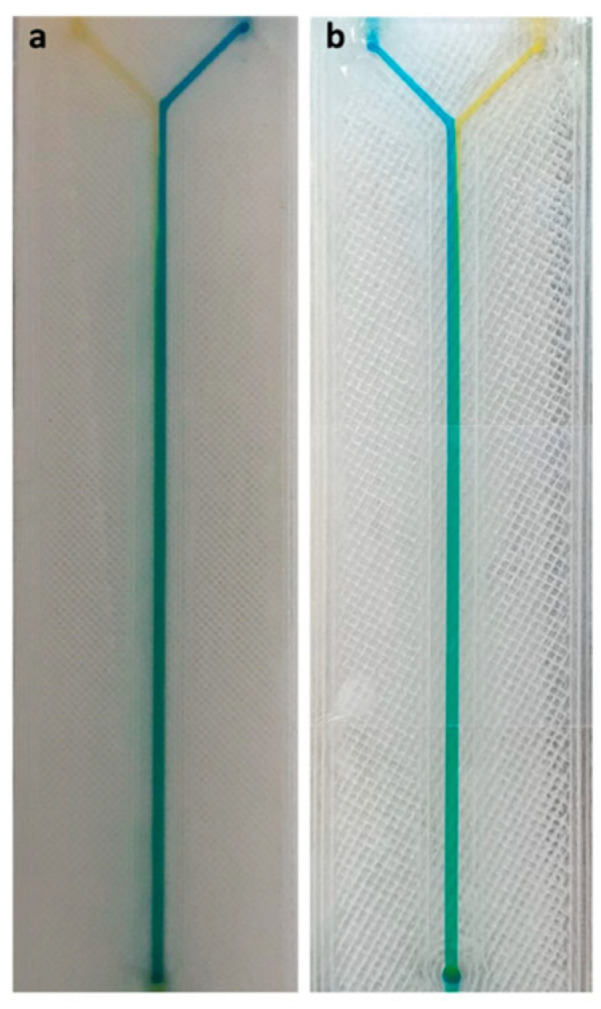
FFF-printed micromixers, whose channels were filled with different colourants, to verify the mixing power of the device. The (**a**) model was made in translucent PLA, the (**b**) model was made in transparent PLA. Reprinted from [20].

To better understand the influence of the printing parameters on the formation of ridges and, in general, on the mixing performance of the FFF-printed micromixer devices, they also performed a deeper investigation on them, in reference [20]. They fabricated the same devices described above, using two different PLA materials from Fabbrix, transparent PLA and translucent PLA, and two different printers, the Ultimaker 3 and the Ultimaker S5, from Ultimaker (Figure 9). The characteristics of the printers were as follows: a layer resolution of 20–200 µm in the Ultimaker 3, and 60–150 µm and 20–200 µm in the Ultimaker S5, based on the nozzle used, 0.25 mm or 0.4 mm, and an accuracy along three axes of 12.5 µm, 12.5 µm, and 2.5 µm for the Ultimaker 3 and, and 6.9 µm, 6.9 µm, and 2.5 µm for Ultimaker S5. During the printing phase, some parameters were fixed, like the printing temperature (190 °C for the 0.25 mm nozzle and 200 °C for the 0.4 mm nozzle), printing speed (30 mm*s^−1^ for the 0.25 mm nozzle and 70 mm*s^−1^ for the 0.4 mm nozzle), layer height (100 µm for the 0.25 mm nozzle and 100 µm for the 0.4 mm nozzle), orientation (60° for the 0.25 mm nozzle and 60° for the 0.4 mm nozzle), flow (100% for the 0.25 mm nozzle and 100% for the 0.4 mm nozzle), infill line distance (0.2 mm for the 0.25 mm nozzle and 0.6 mm for the 0.4 mm nozzle), and built plate temperature (60 °C for the 0.25 mm nozzle and 60 °C for the 0.4 mm nozzle), while the others were varied and their influence on the mixing performances evaluated. Changing the flow rate from 50 µL*min^−1^ to 100 µL*min^−1^, they observed that the complete mix length increased, while increasing the line width from 200 µm to 600 µm, the length for a complete mix decreased. The different materials did not have an influence on the mixing performances, because all of them are hydrophobic, but they influenced the possibility of monitoring the fluid flow, due to the opacity of translucent PLA and the semi-transparency of transparent PLA, which required back illumination to perform imaging techniques. Between the two selected printers, the Ultimaker S5, which had better accuracy, ensured devices with better mixing performances [20].

### 3.3. Material Jetting

#### 3.3.1. Multi-Jet Modelling/PolyJet

Nowadays, PolyJet technology, developed by Stratasys, is widely used in the MEMS field for different applications, like actuator fabrication or the microfluidic device replica moulding process.

**Figure 10:**
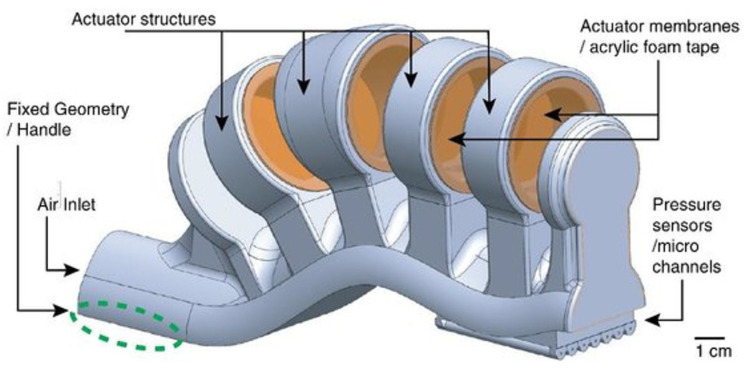
Polyjet-printed actuator; functional parts are indicated by arrows: structural elements, inlets and outlets for air flux, pressure sensors, and channels for EGaIn. Reprinted from Flexible and Printed Electronics, vol. 4, no. 3, F. Spina, A. Pouryazdan, J. C. Costa, L. P. Cuspinera, and N. Münzenrieder, “Directly 3D-printed monolithic soft robotic gripper with liquid metal microchannels for tactile sensing,” August 2019, with the permission of IOP Publishing, Ltd. [2].

The first example here reported comes from the group of Spina et al. [2]; they explored the possibility of directly 3D printing a soft robotic actuator that integrated a pressure sensor, for tactile and gripping applications. Their inspiration came from living organisms that show a proprioceptive ability; so, they tried to replicate it in an artificial finger that generated force and provided feedback about pressure, through a series of microchannels where a liquid metal, eutectic gallium–indium (EGaIn), flowed with a certain resistance that varied with pressure. This device was conceived from the perspective of collaborative manufacturing for middle-sized parts. The printed object, whose CAD rendering is reported in Figure 10, was fabricated with Objet30 Prime^®^, from Stratasys, a PolyJet printer with a 28 µm resolution, using as a printing material the TangoBlack^®^ FLX973 rubber-like acrylic and, as a support material, SUP706, both from Stratasys. The microchannels were seven in number, with a diameter of 350 µm each, and they were connected to the electronic readout for a real-time monitoring of the pressure value during the gripping phase. The actuator part was composed of a ring-shaped structure mounted over a hollow main body, pneumatically actuated, so the insertion of the air inside the main channel provoked the expansion of the wall, which meant the deflection and bending of the whole structure.

The initial air pressure controlled the bending angle, and, simultaneously, the sensors determined the pressure applied during the grip, that could be up to 900 kPa. This scheme was designed to better distribute the stiffer region on the main body and the less stiff one on the ring, and to achieve a better mechanical resistance to deformations, because the material mechanical properties were quite low. The performed mechanical tests revealed that the maximum bending angle for the structure was 12°, with an actuating pressure of 16 kPa, to avoid damage and fractures to the material, but if the bending radius became <1.2 cm, there was permanent damage to the devices, in particular, to the sensor part. The sensor detected up to 900 kPa, sensing the hydraulic resistance variation and, through it, the electrical variation. The material showed a high resistivity, so the power consumption was low, while the sensor lifetime was significantly high, up to 48 h, even if the sensitivity was lower compared to the state of the art. The sensor’s working frequency was limited to 3.6 Hz, linked to the printing material and channels geometry, but it was sufficient to cooperate with the 0.3 Hz actuator with which it was paired, in order to efficiently control the sliding and gripping of the elements, with a 2.5 N gripping force and 16 kPa actuating pressure guaranteed [2].

Another MEMS actuator fabricated through PolyJet technology is reported in the work of Savaş et al. [8]; they presented a scan head for laser-scanning endoscopy, 45° tilted with respect to the probe axis. They tested three head types, a 10 mm × 10 mm commercial one, a 10 mm × 10 mm 3D-printed tilted one, and 5 mm × 5 mm 3D-printed tilted one. Because of the small dimensions and high accuracy and resolution required, they chose to print the scan heads using a mix of the Stratasys materials, Veroclear and TangoBlack (Stratasys, Eden Prairie, MN, USA), for the 5 mm × 5 mm, and the Veroclear alone for the 10 mm × 10 mm. They evaluated the performances of these devices, to compare them with state-of-the-art MEMS, and they found that the 3D-printed 10 mm × 10 mm, both the conventional and the tilted, showed a total scan angle of 80° for 10^6^ cycles, higher compared to the literature. They also noticed that, due to the higher modal frequencies of the first, second, and third mode of the 5 mm × 5 mm device, the smaller scan head could ensure an SVGA resolution at 10 frames per second (fps) and VGA resolution at 15 fps, which made it suitable for one-time-use endoscopic applications. In their devices, they inserted a silicon reflective mirror reinforced with printed material and a magnet to avoid the deformations that can cause a distortion of images, decreasing the number of resolved spots. Their design proved to be more resistant to distortion, compared to state-of-the-art MEMS, thanks also to the lower scan speed, ensuring at the same time more compactness, due to the 33% of volume reduction achieved [8].

As with the other technologies presented above, PolyJet/MJM has been proposed in the literature as a fabrication technique for microfluidic devices that have been directly 3D-printed or fabricated through the replica moulding of a 3D-printed mould.

A first example of this second type of application is reported in the work of Cairone et al. [49]; they fabricated a micro-optofluidic switch, using a T-junction coupled with three channels for the optical fibres. In a micro-optofluidic switch, a laser beam is reflected and transmitted selectively through a channel filled with a two-phase flow; in this study, they were air and water. These two materials show different refractive indexes, and the laser impacts on the fluid surface with a well-defined inclination angle, and it is transmitted or reflected following Snell’s law, which allows one to understand the final displacement of the light, considering the geometrical parameters, like the channel dimensions, together with the optical ones. The device dimensions were the following: the distance between the inlet and the junction was 13 mm, the inlet and outlet diameter was 2.4 mm, the channel width was 420 µm, then 250 µm, and the channel height was 520 µm, then 350 µm. They fabricated and tested two versions, and both moulds were 3D-printed using, as a printer, Objet 30, from Stratasys, because it had a resolution of 49 µm in the plane and 28 µm out of the plane along the Z axis and the accuracy was 0.1 mm, while they selected as the printing material and support material Whiteplus 835 and FullCure 705 (Stratasys, Eden Prairie, MN, USA), respectively. After the functionalisation step of the moulds with Sokalan to enhance the PDMS peeling off, they replicated them using a PDMS precursor and curing agent mixed in a ratio of 10:1. The tests were conducted in static and dynamic conditions, and the results highlighted the advantages of this device, that was easy to design and fabricate; it did not require lenses or mirrors for the implementation of the optofluidic switch; and it was a low-cost fabrication process, that ensured a device with a good sensitivity and high precision, especially selecting the higher channel diameters [49].

**Figure 11:**
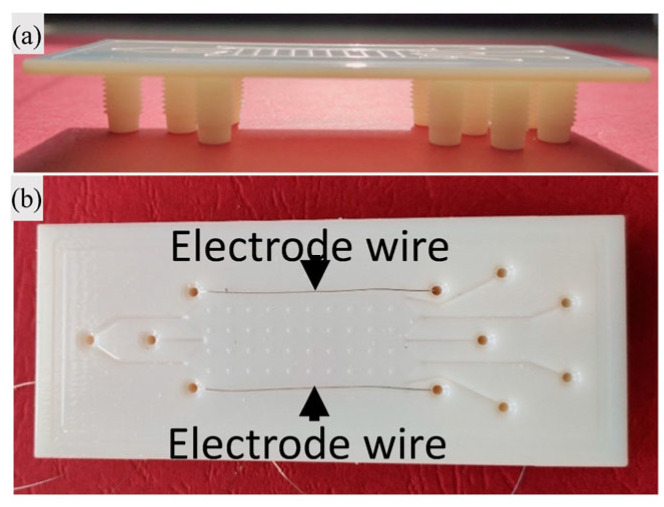
(**a**,**b**) 3D-printed device for micro-FFE application, with stainless steel wire electrode. Reprinted from [50].

An example of a microfluidic device direct 3D-printing fabrication is reported in the work of Barbaresco et al. [50]; they fabricated a chip for the separation of micro- and nanoparticles from a solution using the free-flow electrophoresis (FFE) approach, a versatile method that can segregate species from a continuous flow according to their size and charge. The FFE microfluidic devices include a central separation chamber, with two electrodes for the application of an electric field perpendicular to the chamber, pushing the charged species to deflect during their passage. In this study, they fabricated the device in Figure 11 through a PolyJet 3D printer, the Objet30 from Stratasys, with Verowhiteplus RGD835 as material (materials and printer from Stratasys, Eden Prairie, MN, USA). The microfluidic chamber, whose dimensions were 30 mm length, 13 mm width and 100 µm height, with elliptic pillars of 1 mm × 0.8 mm × 100 µm, was sealed using a 750 µm-thick PMMA cover, fixed thanks to a glue of PEGDA and Irgacure 819, thermally cured. At the two sides of the chamber, there were two partition bars of 50 µm height, to avoid electrolysis bubbles’ formation due to the strong electric field generated by the two stainless steel wire electrodes in the lateral channels, whose dimensions were 100 µm height and 1.5 mm width. There were two inlet and five outlet ports, whose diameter was 3.2 mm, 500 µm height, on the back of the device, for sample insertion and collection. The device was tested to evaluate the separation potential; so, they concluded that their device was fabricated with a low-cost technology maintaining a 5% dimensional accuracy compared to the CAD dimensions, and it also presented good performances in the accumulation of different species in the tested volume, opening the possibility of micro- and nano-population concentration tuning to a specific buffer through the application of this technique [50].

Another example of a microfluidic device being 3D printed is the work of Donvito et al. [12]; they fabricated a droplet generator using MJM 3D-printing technology. This device, which allows the generation of droplets starting from a continuous phase that flows in the central channel and a second disperse phase injected through lateral one, has six T-junctions organised into a rectangular shape, with the following dimensions: the continuous-phase channel width was 400 µm, the disperse-phase channel width was 200 µm, and both channels’ height was 200 µm. To fabricate this structure, they used the ProJet^®^HD3500 from 3D Systems (Rock Hill, SC, USA), which presents a high resolution of 656 × 656 × 1600 DPI (XYZ) and a layer thickness of 16 µm, while they selected acrylonitrile as the printing material and wax as the sacrificial material. The liquid continuous phase was represented by oleic sunflower seed oil, and the dispersed phase was deionised water coloured with a blue food dye. They tested the ability of this device to generate droplets, and they found the following: it was possible to solve problems linked to misalignment, typical of traditional lithographic techniques, thanks to 3D printing; it was possible to fabricate the complete device in one step and at a lower cost; the final device ensured stable emulsion with small-diameter drops; and the polydispersity index was low, less than 6.3%, which was comparable to the standard fabricated devices, when assessing the equivalency of the selected technology with the commonly applied one [12].

#### 3.3.2. Inkjet Printing

As previously mentioned, pyro-EHD printing technology is rapidly spreading as innovative solution for IJP, for its simplicity, flexibility, high resolution, versatility, both in the printing materials and in substrates, and its free-from and nozzle-free characteristics.

A first application of pyro-EHD to device fabrication is represented by the work of Coppola et al. [24]; they fabricated polymeric microlenses inside a pre-existing microfluidic device in PMMA and on top of fibre terminations. Their pyro-EHD system is the same as reported above in Figure 1, with a microscope glass slide as the liquid reservoir base, in direct contact with a z-cut and 500 µm-thick LN crystal; a cover slide treated with hydrophobic tetra-ethylorthosilicate/1H,1H,2H,2H-perfluorodecyl-triethoxysilane (TEOS/PFTEOS) as the receiving substrate; a moving stage with a high-precision linear motor with a maximum travel speed of 30 mm*s^−1^; and a monitoring system with a high-resolution (2048 × 2048 pixels) camera and a 470 nm LED 650 mW power light source. The material used for the microlenses printing process was PDMS, firstly printed on a cover slip, then into the channel of the PMMA microfluidic device, obtaining arrays of separate droplets controlled in the diameter dimension, from 50 µm to 700 µm. Moreover, they printed a biopolymer on top of the fibres, creating a pattern for controlling cell adhesion and spread among them, and, concurrently, they used the same biopolymer to print a micro-axicon lens inside a lab-on-a-chip, to obtain a final device that can change the incident beam shape. They tested their devices using an interferometric Mach–Zehnder interferometer in the transmission configuration to perform a digital holography of the microlenses. They concluded that this technique allowed them to work in a stable droplet generation condition, helping the fabrication of freeform 3D structures and overcoming the limitations linked to the printing material, the substrates, the resolution desired, the nozzle dimension rescaling, and their clogging phenomenon [24].

A second example of a pyro-EHD application for microlenses fabrication is the work of Mandracchia et al. [51], which fabricated a pocket module for holographic microscopy using a lab-on-a-chip. They tried to move the complex apparatus required for holography, like interferometers and so on, directly into the chip, inserting optical features such as a grating and microlenses for extracting the reference wavefront and the hologram fringes. The chosen microfluidic device was a commercial one, while for the grating fabrication, they relied on the interference lithography of a positive photoresist, the Microresist ma-P 1210, and for the microlenses, they used the described pyro-EHD apparatus, obtaining the polymeric lenses inside the channel. They tested the performance of the as-fabricated device, using a single collimated beam to irradiate the channels and collecting the outgoing beam, the object wavefront, that interacted with static samples or flows inside the channel, and the reference, that was diffracted by and interacted with the previous one. They coupled the physical device with a linear sensor array to enlarge the field of view and used a space–time digital holography technique as an elaboration protocol for the images, increasing the throughput of the microscopy technique [51].

### 3.4. Powder Bed Fusion

#### 3.4.1. Micro Metal Laser Sintering

MLS is one of the more widely used AM technologies for metals, because it enables one to produce large amounts of parts in a one-step process that requires few post-processing treatments, and it is one of the few technologies that allow the stacking of printing elements.

This is the reason why the group of Galati et al. [52] proposed the use of direct MLS for the redesign and fabrication of a flying measure probe for MEMS testing. The aim of their work was to re-think the support and moving system for the flying probe, to improve the accuracy of measurements and to reduce the weight and the number of the printed parts maintaining the stiffness, and to fabricate it through a high-resolution and dimensional-accuracy technology. The total structure dimensions were 220 mm × 690 mm × 101 mm, and it was composed of the probe; a support bracket, which contained the vision system and the lighting system for the images acquisition; the probe moving system; and the data collection systems. The probe could move along the X and Y axes thanks to a motor, and it could also move along the Z axis. The whole system was composed of 16 different elements that had to be assembled, so they tried to reduce the number of elements to 3, which were printed and then assembled. The need for elements reduction is linked to the geometric errors that appeared, accumulated, and propagated, due to the machining of the metal with a three-axis milling machine. Their probe redesign focused on the X- and Y-rails, that allowed movement along the two axes, and the bracket. The adopted solution was to replace the vertical walls with thin honeycomb-structure walls to reduce weight, to efficiently redistribute stress, and to obtain a self-supporting structure. The structures were printed using, as printer, an EOS M400 machine, from EOS (Kraillig, Germany), and gas-atomised AlSi10Mg powder as a printing material. The printed parts were detached from the building platform through EDM, so they did not require any finishing approach for the surfaces. The total weight of the final part was reduced by 32%; the parts were also 50% stiffer compared to the original ones, saving a significant amount of material at the same time, with a positive impact on the environment [52].

**Figure 12:**
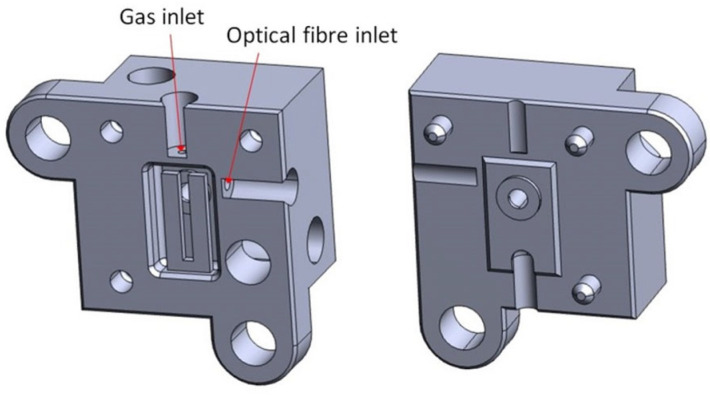
The two monolithic stainless steel parts of the TFEPAS ADM, with the two small apertures evidenced with red arrows: one opening was for the optical fibre insertion, the other was for the gas sample to analyse. Reprinted from [53].

Another interesting application of MLS is presented in the work of Viola et al. [53]; they fabricated an absorption detection module (ADM) for Tuning Fork-Enhanced Photo–Acoustic Spectroscopy (TFEPAS) through µMLS. TFEPAS is an analytical technique that involves a non-piezoelectric tuning fork and an interferometric optical readout to measure the photoacoustic excitation of the tuning fork. They printed an assembly, whose two parts are represented in Figure 12, in stainless steel that comprised the ADM, the fork, and the acoustic micro-resonator, to obtain a final TFEPAS that was customisable without the need for manual alignment of the various elements, and was miniaturised and thermal resistant, thanks to the absence of welds and the elimination of thermal mismatches between materials. The assembly was printed in two different parts to assemble, allowing a visual inspection of the fork before they were closed together. The designed fork required a small size and high spatial resolution, and they fabricated it, thanks to a Swiss company, Precipart (Lyss, Switzerland), with the following dimensions: 4 mm tines length, 0.75 mm tines width, 0.4 mm tines thickness, and 0.5 mm tines distance. The micro-resonator, with an internal diameter of 0.9 mm, was positioned 0.1 mm behind the fork, to obtain a photoacoustic coupling, avoiding high damping phenomena. All the surfaces had to be polished to maximise reflection, so they polished them mechanically. Then, they inserted the monolithic body into the readout setup, which included a HeNe laser, a photodiode, a cube beam splitter, supports and micro-manipulators for precision positioning, and a quantum cascade laser for stimulating the fork’s vibrations. Last, they evaluated the performance of the device as a gas analyser, using ammonia, and the results showed that the limit of detection was under 1 ppm, with a good identification potential through the accurate measurement of the infrared fingerprint. So, they demonstrated that this solution could ensure a good alignment between the tuning fork and the acoustic micro-resonators that were printed monolithically, the miniaturisation of the internal volume, and the possibility of working at high temperatures, maintaining low costs and the customisation of the tuning fork [53].

#### 3.4.2. Selective Laser Melting

SLM technology has been pushed in recent years, due to the increasing demand for smaller and smaller mechanical components for MEMS and NEMS devices.

An example of a MEMS accelerometer being SLM printed is reported in the thesis of Corigliano [54], which proposed a biaxial accelerometer and a capacitive one using the titanium alloy Ti-6Al-4V and the Renishaw AM250 printer (Wotton-under-Edge, UK), whose printing chamber was at 150 °C in a 21 mBar argon atmosphere. For the biaxial accelerometer, the design included four simple folded beam springs, one for each side, to detect acceleration along X and Y axes, with two different resonant mode frequencies, 420 Hz and 400 Hz. The accelerometer’s total area was 20 mm × 20 mm, with an inner spring thickness of 0.5 mm and outer spring thickness of 1 mm, due to the printer’s minimal feature-size limitations. For the capacitive accelerometer, the design included a suspended mass linked to four springs, with a voltage applied between it and conductive plates to read the capacitance variation due to the mass displacement. This design presented some constraints to consider, such as the need for an electrically insulating layer between the conductive ones; so, they had to print the sensor as two elements that were then assembled, interposing a PVC tape. The capacitive accelerometer had a volume of 15 mm × 15 mm × 15 mm, with a minimum feature thickness of 0.5 mm for the springs. The parts were printed, inserting supports under them to anchor them on the printing plate, and the supports were removed manually. A characterisation of the printed parts highlighted that the printed parts had a thickness reduction of 0.15 mm compared to the CAD dimensions; they suffered from thermal deformation, which could be reduced with heat treatment and a redesign that involved a more symmetrical architecture; their devices also showed low sensitivity and a certain level of surface roughness [54].

Therefore, the SLM’s printed parts quality, i.e., their surface roughness and porosity, is heavily affected by some phenomena, like the stair step effect, linked to the approximation of curves to a straight line during layer-by-layer deposition, and the balling effect, small spherical agglomerates of melted powder that cause the formation of discontinuous lines. To find a solution to these defects that lower the quality of printed mechanical parts, the group of Hassanin et al. [55] studied the effect of micro-EDM on the surface roughness and porosity of micro-gears fabricated through SLM technology. First, they printed the parts using a titanium alloy (Ti-6Al-4V) as a printing material and the Concept Laser M2 powder bed system as a printer, in an inert atmosphere thanks to an argon flux inside the chamber. The printed parameters were optimised, evaluating the porosity level of the printed parts, and the final parameters combination that ensured a lower porosity level was 200 W for the laser power, 1000 mm*s^−1^ for the scan speed, and 0.67 for the hatch spacing, to ensure an energy density of 100 J*mm^−3^ and a porosity of 0.25%. Concerning the surface roughness, the initial registered value was 14.7 mm, so they performed an optimisation process for micro-EDM parameters, to find the ideal parameters for lowering it. Thanks to this technology, they obtained a final roughness of 4.6 mm and a finishing regime between 0.6 mm and 0.8 mm, improving the surface quality due to the reduction of surface discharge energy, taking from mechanical micro-components with better quality and mechanical performances [55].

## 4. Materials for AM Fabrication of MEMS

MEMS technologies originally developed from the semiconductor industry; thus, silicon is the primary material used in the manufacturing MEMS devices [56]. Silicon has been the gold standard as a MEMS material, due to its mechanical, thermal, and electrical properties. Indeed, silicon has almost the same Young’s modulus as steel, but is as light as aluminium, and in addition, it has a similar thermal conductivity to metals but a lower coefficient [57].

On the other hand, silicon presents some drawbacks: Si-based MEMS devices cannot operate at a temperature above about 200 °C due to the degradation of pn-junctions, and the elastic modulus drastically drops above 600 °C [58]. These characteristics are limitations for high-temperature applications. In addition, silicon is intrinsically brittle, so not ideal for applications where flexibility is required, e.g., smart skin for tactile uses and flow sensing [59,60].

Therefore, new materials such as compound semiconductors, ceramics, and polymers have been evermore incorporated in MEMS production processes, to enhance their functionality, performance, or fabrication simplicity [57]. Generally, MEMS devices have been fabricated using renowned micromachining techniques, such as photolithography, oxidation, sputtering, thin-film deposition, etc. [61]. This aspect has been already reviewed in the literature, and it is outside the scope of this manuscript [3,62]. Herein, the discussion will be limited to the materials used to produce MEMS through AM techniques.

### 4.1. Polymers

Polymers have been used to replace silicon in MEMS fabrication since they possess some features almost complementary to silicon, such as flexibility, easy processing, insulation, chemical and biological functionalities, biocompatibility, and a low cost [63].

These properties have enabled researchers to broaden the fields of application, out of more well-established fields such as sensors, actuators, or microsystems applied in the aerospace, automotive, or military fields [63,64]. Indeed, polymer micromachining technologies are employed in the biomedical field, as a micro-total analysis system, labs-on-a-chip [65], in drug synthesis [66], drug delivery [67] and so on; these technologies are also known as Bio-MEMS.

In the literature, many polymers are reported for MEMS/Bio-MEMS, e.g., SU-8, which is a commonly used epoxy-based negative photoresist, polydimethylsiloxane (PDMS), and polymethyl methacrylate (PMMA).

SU-8 is widely used in MEMS/Bio-MEMS production, basically with traditional fabrication methods, due to its ability to form high-aspect ratio structures. Among its most prominent features are biocompatibility, mechanical stability, transparency to UV light and chemical resistance to most solvents, once crosslinked [68,69].

Despite the advantages of SU-8, several disadvantages are reported, such as delamination of the microstructure and film [70], self-fluorescence, which leads to background noise in the case of optical measurements, and the surface hydrophobicity which makes it necessary to modify the surface chemistry to make SU-8 compatible for bio-applications like microchannels or cell sorters [68].

SU-8 can be used as the main material for the fabrication of MEMS devices; for example, Bernasconi et al. [71] designed a fully inkjet-printed electrochemical impedance spectroscopy (EIS) probe in order to detect the presence of cancerous cells, evaluating the variations in the state of the cells in term of the presence of metabolic toxicity or perfusion loss. In this work, SU-8 is inkjet-printed to realise the main body of the probe and to pattern pads and tracks, realised with platinum (Pt), which is patterned inside an SU-8 printed mask employing electrodeposition. The device was tested to evaluate the bioimpedance of different animal tissues, demonstrating the actual functionality of the device.

PMMA is a transparent low-cost polymer with a very low auto-fluorescence over a wide spectral range. Due to these features, PMMA has been commonly used for various biochemical separations, such as in electrophoresis and DNA sequencing. In contrast to silicon or glass, which require a high temperature for bonding, PMMA can be bonded at a lower temperature; this makes the fabrication of PMMA-based MEMS devices low-thermal budget and less expensive [65,68]. Despite PMMA showing some features which make it suitable, especially for biomedical applications, to date in the Italian scenario—unlike other foreign research groups, where PMMA is used as the main material for the fabrication of MEMS devices [72]—PMMA is still mainly used as a mould for the fabrication of microdevices such as microfluidic devices [73] or other applications such as micromixers, which are essential components in microfluidic devices, since they have a direct impact on the efficiency and sensitivity of assays [74].

PDMS is an elastomeric polymer which has specific properties for biomedical ap-plications, including resistance to biodegradation, biocompatibility, chemical stability, and gas permeability. Moreover, it is generally inert, non-toxic, and non-flammable [75]. All these features have made PDMS one of the most widely used materials in the manufacturing of microfluidic devices for the development of systems relating to areas such as drug delivery, DNA sequencing, clinical diagnostics, etc. In addition, PDMS has been commonly used in the fabrication of biomodels [76].

Moreover, its surface properties can be changed in order to obtain tailored properties. In the literature, many works use PDMS, particularly for the fabrication of microfluidic devices exploiting traditional fabrication methods like bulk/surface micromachining, wafer bonding, lithography, and other micro-processing techniques [34].

Although these techniques are well-proven, the recent trend is the one-step direct 3D printing of micro-devices. Nevertheless, some 3D-printing processes, such as inkjet 3D printers, require materials which are either cytotoxic or non-transparent and which are not consistent with the characteristics of microfluidic devices. Therefore, PDMS is the gold standard for this application, but unfortunately, it is still not compatible with many 3D-printing techniques.

So Cairone et al. [77] present a PDMS micro-optofluidic device, realised by the master–slave 3D printing approach, which aims to overcome this limitation and to extend the use of 3D printing in the realisation of micro-optical components. The proposed device was designed to be a compact device that integrates micro-optic and micro-fluidic components, and it was used both for chemical fluids and cells detection, by exploiting the different light transmission correlated to fluid interfering with the laser beam in a microchannel section.

**Figure 13:**
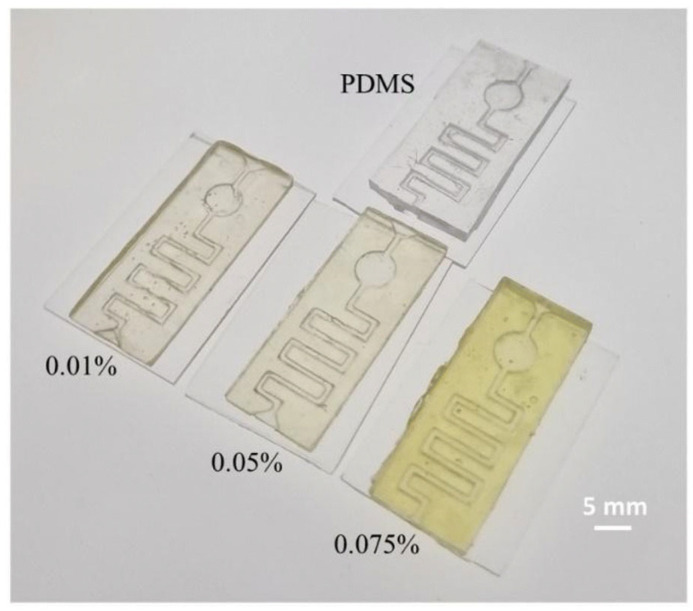
Picture of PDMS and 3D-printed acrylate PDMS 0.01–0.05–0.075% dye microfluidics devices. Reprint from [78].

Other PDMS-like materials have been 3D printed, with chemical functionalities suitable for other AM technologies. As mentioned before, Gonzalez et al. [39] developed fully 3D-printed microfluidic devices based on a 3D-printable PDMS-based acrylate (TEGORad), achieving channels of 300 µm. Starting from this study, Villata et al. [78] developed a device for cell culture and drug testing (Figure 13). TEGORad is an acrylate PDMS copolymer which can be 3D printed through DLP. This material showed easy and fast printing, allowing the production of complex microfluidic devices, keeping the prominent features of PDMS, such as transparency, flexibility, and chemical properties. In addition, the in vitro study shows that TEGORad can sustain cell growth without causing any genotoxic effect.

Another important polymer that can replace PDMS for the fabrication of microdevices is Polylactic Acid (PLA). PLA is a biocompatible and bioabsorbable thermoplastic material derived from renewable plant resources [79]. It can degrade after use in CO_2_ and water, so it can be adopted for a sustainability approach. In shows excellent mechanical and piezoelectric properties in the form of cellular foams, and it can be easily 3D printed by FFF printing. Ongaro et al. [79] proved the suitability of PLA as a substitute for PDMS, especially for microfluidic cell cultures and organ-on-chip applications. PLA has been demonstrated to be transparent, suitable for stable functionalisation, biocompatible, and without absorpting small molecules. Perna et al. [80], instead, investigated the piezoelectric performance of the material. This study shows the potentiality of designing a biocompatible and sustainable piezoelectric device for energy-harvesting and biosensing applications.

Tiboni et al. [66], for example, developed a 3D-printed microfluidic chip through FFF to formulate ethanolic liposomes loaded with 18-α-Glycyrrhetinic acid (GA), which is a bioactive compound that exhibits many biological and pharmacological effects. Indeed, since a microfluidic chip offers a great control of micromixing under a laminar flow, it presents an interesting alternative for the preparation of lipid-based nanovesicles.

In this work, they were able to create an inexpensive and easily scalable microfluidic device that can produce liposomal dispersion, with good physicochemical characteristics and an EE% of GA up to 63%.

One of the most important characteristics of polymeric materials is the ability to easily functionalise the backbone chain or combine, to modify and tailor materials properties to provide new functionalities.

For example, Roppolo et al. [81] developed a new functional 3D-printable material suitable for DLP printers, exploiting photocurable monomers and a photoactive dye (a molecule with azobenzene moieties) that give a light-triggerable and reversible mechanical response, since the incorporation of azobenzene units into polymeric matrices enables a reversible and controllable change in the Young’s modulus of a 3D printed microstructure upon laser irradiation. They printed micro-cantilevers in single 3D-printing step, which could have many interesting applications in the MEMS field.

In another work, Gillono et al. [82] exploited the photoactivity of azobenzene chromophores to develop a material presenting light-triggered CO_2_ permeability. First, they tested the light-induced changes in CO_2_ upon green light irradiation of a resin with poly (ethylene glycol) diacrylate (PEGDA), with different azobenzene chromophores. Finally, as a proof of concept, 3D structures presenting light-triggered CO_2_ permeability have been built.

**Figure 14:**
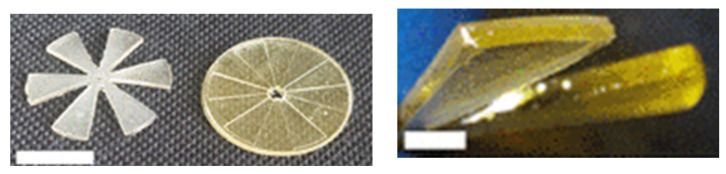
Image of 3D-printed rotor and stator components of rotating TENG and rotor blades showing PEGDA/TEGORAD layers. Reprinted from [83].

Chiappone et al. [83] studied the application of the DLP technique to the 3D printing of triboelectric nanogenerators (TENGs). First, they tested different photocurable resins for DLP printing as triboelectric layers in a contact–separation triboelectric nanogenerator configuration, using TEGORAD, as the most tribonegative material, and EB4740, a polyurethane acrylate, as the most tribopositive one. Once the best-performing couple of materials had been found, they fabricated 3D-printed TENGs devices using a multi-material printing procedure with increasing geometrical complexity (Figure 14). The TENGs devices show the ability to recover energy from different kinds of mechanical movement.

**Figure 15:**
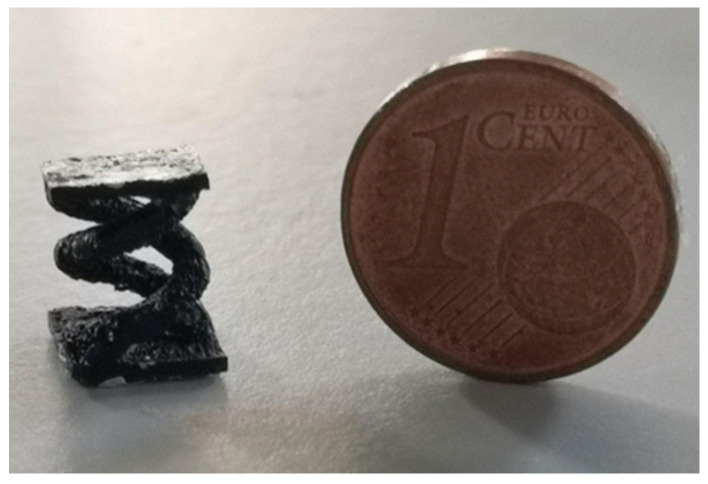
Image of 3D-printed double-helical sample from PEGDA:PEDOT resin. Reprinted from [84].

Scordo et al. [84] developed a resin that was 3D printable by means of SL, based on poly (ethylene glycol) diacrylate (PEGDA) and PEDOT: PSS, in order to obtain conductive 3D objects (Figure 15) which can interact with chemical vapours over an extended period, by ensuring a reliable alteration in their structural and conductive characteristics.

### 4.2. Ceramics

For high-temperature application, Si-based materials, as well as polymers and metals, are limited by their characteristics, since they cannot withstand temperatures greater than a few hundred degrees Celsius (the softening temperature of Si is 600 °C) [85]. In addition, silicon is not a structural material, since it has a fracture toughness of almost 0.7 MPa*m^1/2^ and a high degree of reactivity to oxygen and water. Thus, for these reasons, many features of ceramics, such as low density, high-temperature mechanical properties, chemical inertness, and resistance to corrosion at high temperatures, make it suitable for several MEMS applications, such as chemical engineering, microsensors, biomedical, nano turbines, etc, especially in harsh environments [86]. To take advantage of the well-known characteristics of silicon, it is first worth considering Si-based ceramics.

In particular, silicon carbide (SiC) is considered to be a semiconductor with excellent physical and chemical characteristics with respect to Si, including a wide bandgap, mechanical strength, high thermal conductivity, high melting point, and inertness to exposure in corrosive environments [87]. In addition, SiC has a Young’s modulus (700 GPa) higher than Si (190 GPa) [58].

SiC materials have a variety of different crystal structures, but although more than 250 polytypes have been found to date, only three crystalline structures exist: cubic, hexagonal, and rhombohedral. The atomic composition is the same in all SiC polytypes; nevertheless, they differ, especially in terms of electrical properties such as band gap or electron mobility [88]. This material has played a key role in the advancement of modern semiconductor technology, specifically for lightweight/high-strength structures, nano-biomedical devices, optical elements, and high-temperature semiconducting devices [89].

SiC is fabricated by the solid-state reaction between petroleum coke and SiO_2_ at 2000 °C; however, this traditional fabrication method is high in energy consumption; thus, it was necessary to introduce a low-energy fabrication method. So, from the early 1970s, organosilicon polymers have been used to fabricate silicon-based ceramics [90]. Organosilicon polymer, also known as silicon-based polymer-derived ceramics (PDCs) are prepared by the controlled pyrolysis at relatively low temperatures of preceramic inorganic–organic hybrid polymers containing Si, H, C, O, N, and also other elements such as B, which are grafted to the silicon atoms for the formation of different organo-silicon polymers such as polysilane, polycarbosilane, polysilazane, and polysiloxane [90,91]. This process leads to the creation of a nanostructured ceramic that can be controlled through polymer chemistry, polymer structure, and the ceramitisation process [92].

The design of the molecular precursor plays a significant role in determining the macroscopic chemical and physical properties of PDCs [93].

For this reason, these materials have excellent thermal stability, oxidation/corrosion resistance, and creep resistance, which make them suitable for high-temperature sensors [94], MEMS devices, conductivity coating [95], biomedical application [96], and so on.

Silicon carbonitride (SiCN) and silicon oxycarbide (SiOC) are two of the most well-known systems of PDCs, since they have shown great potential in many different applications. AM processes are the best choice to fabricate complex structured materials with a high printing resolution starting from a CAD. Concerning ceramic materials, many AM processes have been exploited to fabricate 3D microstructures. However, the only viable way to get a combination of the resolution and surface smoothness of a 3D micro-component, typical of polymer-based techniques, with the chemical, mechanical, and thermal properties of ceramic materials, is by fabricating 3D ceramic microparts using an STL of PDCs [97].

**Figure 16:**
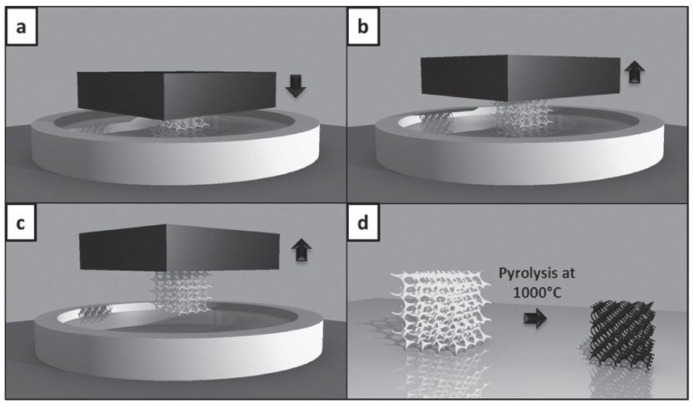
Additive manufacturing of the preceramic photopolymer: From the printing process to the pyrolysis process of the preceramic 3D component: (**a**) the moving stage is lowered to a gap height corresponding to the height of the layer to be cured and (**b**) moves upward to release the component from the vat, before lowering again to continue the layer-by-layer fabrication. (**c**) When the 3D component is completed, it is removed from the moving stage. (**d**) The SiOC ceramic microcomponent (black in figure) is obtained after pyrolysis at 1000 °C of the preceramic 3D component (gray in figure). Reprinted from Advanced Materials, vol. 28, no. 2, E. Zanchetta, M. Cataldo, G. Franchin, M. Schwentenwein, J. Homa, G. Brusatin, and P. Colombo, “Stereolithography of SiOC Ceramic Microcomponents,” January 2016, With permission from John Wiley and Sons [97].

For example, Zanchetta et al. [97] fabricated a 3D-direct ceramic component from preceramic poly-siloxanes with a high ceramic yield, using a lithography-based ceramic manufacturing (LCM) process (Figure 16). Through the LCM technique, they obtained dense and crack-free 3D SiOC micro-components through the pyrolysis of the crosslinked green body at 1000 °C, using a chemically modified, commercially available, preceramic photosensitive methyl-silsequioxane polymer.

In another work, Zocca et al. [98] fabricated a ceramic device owning an order of porosity by the powder-based 3D printing of a preceramic polymer, introducing the crosslinking catalyst either by mixing it with the preceramic polymer or with the printing liquid solvent. Finally, a micro-CT analysis of the printed components showed the printability and the precision of the printing procedure with respect to the starting CAD model.

### 4.3. Metals

Metals are another relevant category of materials for MEMS fabrication. Thin metal films are a part of microelectronics technology; they are used to form conductors, contacts, and reflecting coatings; in addition, they are often used as a structural layer or as sacrificial or protective layers [99,100]. For example, for electrochemical sensor applications, metals are still the main material class, especially for their excellent electrical conductivity, mechanical stability, and versatile chemical functionality [100]. Another important application is for radio frequency (RF) MEMS. Indeed, silicon or highly doped silicon resistivity is too high for most radio frequency (RF) MEMS with respect to metals and, in addition, metal micromachining does not require as high a processing temperature as does silicon technology. Thus, metals are suitable materials for these applications [101].

It is important to consider two fundamental criteria to choose the correct material for these applications: low contact resistance and high reliability [102].

The most common material used as an electrode in electrochemical sensors is gold (Au). Indeed, Au is easily deformed, has a high melting point, is efficient in propagating RF signals, is corrosion resistant, has low resistivity, and is easily deposited [103].

Another important metal is aluminium (Al), due to its high conductivity, chemical stability, linear response, and easy deposition [104,105].

Platinum (Pt), as well as Al and Au, has been used in the MEMS field, since it has a high chemical resistance, high melting point, and high Young’s modulus. The elevated melting point, in particular, enables the construction of structures that exhibit excellent resistance to plastic deformation at high temperature, preserving the conductive properties typical of metals [106]. In addition, Pt receives significant attention for many MEMS devices which work at high-temperature conditions [107].

Silver (Ag) and its alloys are largely used for the fabrication of MEMS, especially for macro-switch electric contact materials, due to their electrical, mechanical, and thermodynamic properties [102]. In addition, Ag, with Au, are the two metals with the lowest absorption in the near-infrared and visible region [108].

The microfabrication of electronic and mechanical structures at the submillimetre scale, including thin-film deposition, photolithographic patterning, and etching, is a time-consuming and expensive process [100,109]. For this reason, an alternative approach was needed to overcome the drawbacks introduced by traditional fabrication processes. Recently, there has been increasing attention towards the fabrication of sensors through printing, owing to its simple implementation and adaptability for large-scale manufacturing [100]. Among the several 3D-printing processes, inkjet printing is one of the leading techniques in AM. Unlike microfabrication processes, the digital nature of inkjet increases the personalisation of products and increases the cost efficiency, since it is a scalable process and because it reduces the generation of environmentally sensitive waste [110].

Metals like Au, Pt, Ag, and Al have been rendered into inks suitable for inkjet printing, which are based on metal nanoparticles. Nanoparticle inks are colloidal liquids, namely, of metal nanoparticles, which can be synthesised by physical or chemical methods and which are suspended either in water or organic solvents [100].

The most common metal nanoparticle inks are made up of Ag, since it has excellent electrical conductivity and resistance to oxidation. For example, Mariotti et al. [111] fabricated the first ever fully inkjet-printed vertically integrated metal–insulator–metal (MIM) capacitors on silicon. They used a Cabot CCI-300 silver nanoparticle ink in order to print a thin-film dielectric layer, and they also printed an SU-8 passivation layer to improve the adhesion of the dielectric layer, since silicon has a surface energy which yields poor wetting. They demonstrated that the final device had a self-resonant frequency (SRF) above 1 GHz and a quality factor (Q) up to 25, which is more than 300% higher than previously reported inkjet-printed MIM capacitors.

Despite all their key features, metals present several drawbacks. For example, Au is a soft metal and has a low melting point, and it adsorbs carbonaceous layers, which make it prone to erosion and wear [102]. Silver and its alloys, for example, stretch to form a nonconductive sulphide layer on the surface which could interfere with good electrical contact and therefore is a problem in MEMS switches [112]. Thus, to overcome these limitations, it is possible to combine several elements to form alloys to obtain the desired characteristics such as low resistivity, high thermal conductivity, no insulating oxides or sulphides, a nominal hardness and elastic modulus, high melting point, and so on [102].

For example, titanium alloys are growingly being considered in mechanical system design, especially in several industries such as the automotive, aerospace, and medical. One of the most commonly used alloys is Ti-6Al-4V, since it has a low density, strong mechanical properties, magnetic resistance as well as elevated temperature performance, a high resistance to corrosion, and high biocompatibility; all these features make it suitable for biomedical or harsh environment applications [113,114].

It is worth saying that the addition of other elements, such as chrome (Cr) or niobium (Nb), in the Ti-6Al-4V alloy enhance the oxidation resistance, respectively at low and elevated temperatures [115]. AM is a promising approach to produce small-sized, complex three-dimensional structures in titanium for MEMS. For example, Zega et al. [116] produced the first uniaxial Ti-6Al-4V alloy accelerometer, fabricated through the laser powder bed fusion (L-PBF) technique, since it allows one to achieve good mechanical properties and a good resolution. The device shows a good performance in terms of sensitivity, so it can overcome the main limitation of polymer-based 3D-printed sensors, since Ti-6Al-4V shows a much better thermal behaviour, which makes it a promising solution for harsh environment applications.

**Figure 17:**
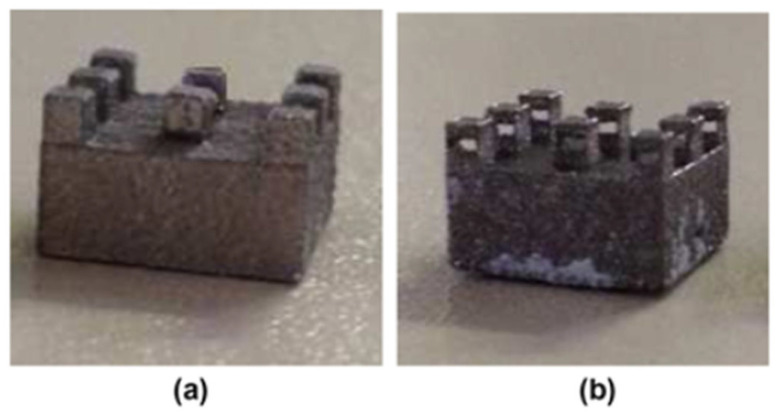
Micro-structured surfaces: (**a**) classical pin fins, (**b**) Venturi-like pin fins. Reprinted from Experimental Thermal and Fluid Science, vol. 55, E. Chiavazzo, L. Ventola, F. Calignano, D. Manfredi, and P. Asinari, “A sensor for direct measurement of small convective heat fluxes: Validation and application to micro-structured surfaces”, May 2014, with permission from Elsevier [118].

Another common material is AlSi_10_Mg, which is a typical casting alloy owing to its high strength/density ratio and thermal properties. In addition, alloying magnesium to a Al-Si alloy allows the precipitation of Mg_2_Si, which enhances the strength of the matrix without compromising the other mechanical properties [117]. For example, Chiavazzo et al. [118] proposed a novel sensor for measuring convective heat transfer on small micro-structured surfaces (Figure 17) made by AM processes, such as direct metal laser sintering (DLMS). This device, due to its features, could be used for the development of a release for industrial applications, elevated temperature measurements, and MEMS.

### 4.4. Carbon NanoTubes (CNTs)

The integration of CNTs into microdevices, in particular for MEMS, offers many exciting opportunities in a wide range of applications as pressure sensors, sensitive accelerometers, or neuronal electrodes, due to their nanometre size, hollow structure, high accessible surface area, low resistance, and high structural stability [119,120]. CNTs also have excellent electrical properties, since they show a high electrons conductivity (up to 109 A/cm^2^) [121] and mechanical properties; indeed, CNTs are amongst the strongest-known fibres, with a Young’s modulus from 270 to 1000 GPa [121,122].

CNTs can be classified basing on their morphology; there exist single-walled CNTs (SWCNTs), double-walled (DWCNTs), and multi-walled (MWCNTs). CNT morphology could influence the electromechanical behaviour of devices, since they present different aspect ratios that directly change electrically conductive networks, which can result in variations in the conductive mechanism [123]. Both SWCNTs and MWCNTs are commonly used for MEMS applications, especially for sensors, actuators, or energy storage devices [122]. CNT integration in MEMS usually entails either bottom-up (in situ growth) or top-down (post-growth) methods. The bottom-up approach involves placing catalytic particles directly onto a substrate to control the positioning of CNTs, while the top-down method concentrates on directing the growth of CNTs to achieve their desired position [124].

The most common manufacturing methods for CNTs are laser transfer, screen printing, and plasma-enhanced chemical vapour deposition. Nevertheless, these manufacturing techniques come with certain challenges, including expense, extended processing du-ration, the requirement for static masks to define specific locations for the nanostructured emitting material, and so on [125]. Thus, 3D-printing methods stand for an alternative method that lend themselves to overcoming these limitations, since they are maskless and allow one to implement hierarchical structures, with features spanning orders of magnitude in size. Indeed, there are many works which are based on printing CNTs and other carbon-based materials to create freeform objects with a high electrical conductivity [125].

An interesting field of research which exploits the key features of CNTs is the development of 3D-printable electrically conductive photopolymers, a polymer nanocomposite filled with CNTs. This nanocomposite material combines the good processability of the polymer part and the high thermal, mechanical and electrical properties of CNTs. Moreover, the addition of CNTs to polymers confers other Joule’s heating capabilities [123].

**Figure 18:**
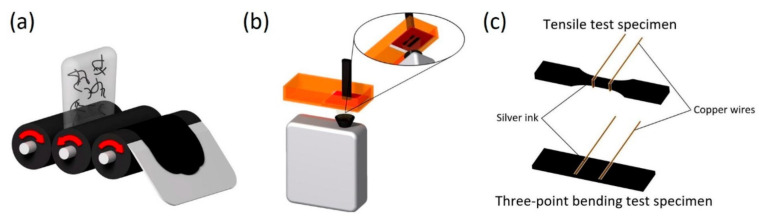
Manufacturing processes of nanocomposite specimens. (**a**) Calendaring dispersion processes, (**b**) DLP 3D-printing process, and (**c**) electrode placement on 3D-printed specimens. Reprinted from [123].

For example, Cortés et al. [123] developed an electromechanical sensing device, based on resins doped with carbon nanotubes, exploiting DLP 3D-printing technology (Figure 18). They used different types of CNT (SWCNTs, DWCNTs, and MWCNTs) to evaluate the effect of their morphology on electrical and electromechanical performance. They studied the effect of the dispersion state of the nanoparticles of three different CNT types with different contents into the matrix, to evaluate their influence on strain sensitivity through electromechanical analysis, and for the crosslinking degree—thus, evaluating the Tg value. First, the dispersion homogeneity is generally better for the MWCNTs, followed by the DWCNTs and SWCNTs, and the degree of the dispersion is enhanced when increasing the CNT content. The dispersion degree, and so the area occupied by CNTs, has a prevalent effect on the curing degree of the material. Indeed, since CNTs are black coloured, they absorb part of the UV radiation from the light source, so they hinder photo-initiator UV absorption, leading to a lower curing degree. Thus, the samples with a higher dispersion degree, which also have a higher percentage of area occupied by CNTs, present lower Tg values and therefore a lower crosslinking degree. These results confirm that SWCNT- and DWCNT-doped nanocomposites show a higher electromechanical performance and a higher crosslinking degree than nanocomposites doped with MWCNTs.

**Figure 19:**
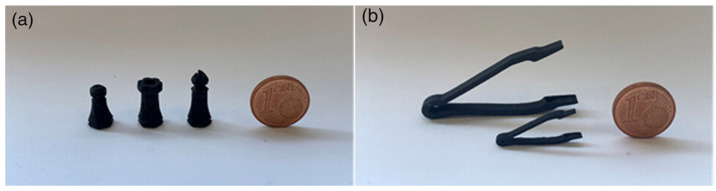
Images of SL 3D-printed (**a**) chess pieces and (**b**) tweezers with different dimensions. Reprinted from Advanced Engineering Materials, vol. 24, no. 12, F. Iervolino, A. Bonessa, G. Foti, M. Levi, and R. Suriano, “Additive Manufacturing of Electrically Conductive Nanocomposites Filled with Carbon Nanotubes,” December 2022, with permission of John Wiley and Sons [126].

Another important work came from Iervolino et al. [126], which developed two photo-curable resins containing MWCNTs toward 3D printing electrically conductive structures using near-visible light (405 nm) stereolithography processes (Figure 19). These two resins belong to two different classes of photopolymers: a free radical system and a hybrid system consisting of a cationic/free radical blend. They first evaluated the rheological properties of the resins by finding that the best is the free radical one, with a concentration of MWCNTs of 0.25%wt. being considered the maximum value to obtain printable inks. In addition, the introduction of MWCNTs results in an average conductivity of 4.2 × 10^−3^ S/cm. This shows the immense potential of this conductive nanocomposite material.

### 4.5. Composite

Despite many materials exhibiting characteristics that can be exploited for various applications, such as conductive polymers that, due to their properties, can be used for electrochemical applications, they are not so close to the ideal performances required, for example, for an efficient electrochemical application. Thus, through composite materials, it is possible to exploit the characteristics of several materials, which, when combined, enable them to achieve tailored properties suitable for a specific application.

For example, Bertana et al. [127] introduced a 3D-printed supercapacitor device employing a 3D-printable electrically conductive polymeric blend based on poly (3,4-ethylenedioxythiophene)-poly (styrene sulfonate) (PEDOT: PSS), exploiting a ceramic substrate. The device was printed with an SL custom SL printer. Different electrode thicknesses were 3D printed to study the scaling properties of the resin, and the several electrodes were electrically and electrochemically investigated.

In another study, Credi et al. [128] proposed a prototype of polymeric cantilever-based magnetic microstructures printed by means of SL. They used Bisphenol A ethoxylate diacrylate as a photopolymer, and to confer ferromagnetic properties to the resin, they used two different approaches: the first was a selective deposition of a metal layer on the surface of the device, the second was the loading of magnetic nanoparticles within the polymer matrix. Finally, they studied the sensing performance of the specimens, in terms of static flexural behaviour versus the magnetic field applied.

**Figure 20:**
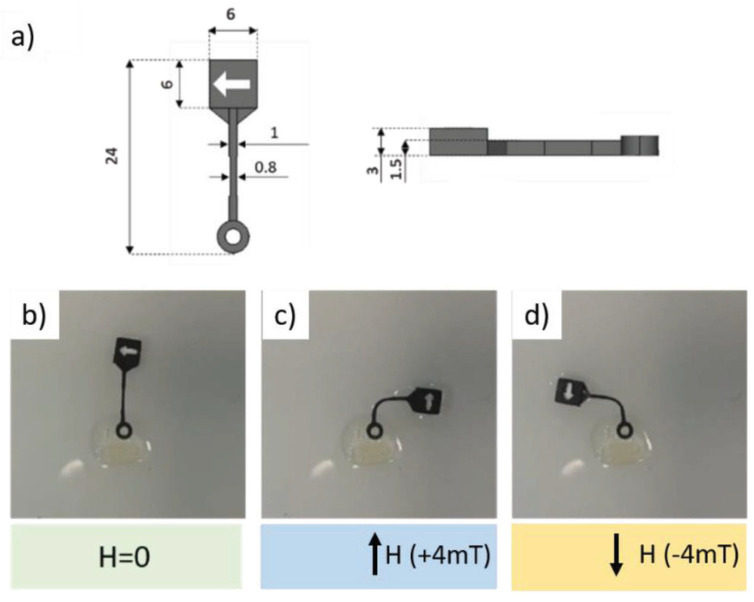
Design of magnetoresponsive soft hammer. Initial configuration and bending of magnetoresponsive soft hammer for two opposite directions of applied magnetic field: (**a**) design of a magnetoresponsive soft hammer. The microstructure is indicated by the arrow on the head of the hammer. (**b**) Initial configuration (*H* = 0) and (**c**,**d**) bending of the magnetoresponsive soft hammer for two opposite directions of the applied magnetic field. Reprinted from [129].

In another study, Lantean et al. [129,130] developed a magnetoresponsive polymer obtained by loading Ebecryl 8232 resin, a urethan-acrylate resin, with Fe_3_O_4_ nanofillers and different amounts of butyl acrylate to control the viscosity of the formulations, the reactivity, and the mechanical properties. In their work, a modified commercial DLP 3D printer was used to obtain hammer-like actuators (Figure 20) that can be successfully used to transfer torque to other gears, thus converting a rotation movement into a linear translation.

## 5. Conclusions and Future Trends

MEMS devices are primarily employed in the electronic, mechanical, automotive, biomedical, and related fields, serving as sensors, actuators, microfluidic systems, and more. Despite the well-established conventional production techniques, there is a demand to reduce production times and costs, consequently increasing production yields. Thus, AM processes represent a valid alternative that addresses these requirements. In this review, the focus was set on the Italian scenario of 3D-printed MEMS, specifically examining works that employ different 3D-printing technologies and incorporate novel and innovative materials. The Italian environment appears particularly vital in this context, being at the forefront of worldwide research, and obviously participating also in international collaborations. Table 2 helps to give a view of Italian research in an international context. The table sums up the possible MEMS applications for the previously mentioned technologies and compares the state of the art of the Italian landscape for 3D-printed MEMS with respect to the international scenario. Various techniques are selected based on their specific characteristics. For instance, SLA is widely utilised because of its precision and high resolution, whereas TPP is frequently chosen for its ability to manufacture nanoscale geometries that cannot be achieved with other manufacturing methods. Material extrusion technologies are also noteworthy for their cost-effectiveness, extensive material options, and the potential to reduce the weight of components. Moreover, material-jetting technologies, including pyro-EHD printing, are rapidly gaining popularity, due to their simplicity, flexibility, high resolution, and versatility in both printing materials and substrates, coupled with features such as being free from nozzles. Finally, powder bed fusion technology includes notable methods such as MLS, extensively employed for metallic components, and SLM, which can be used for manufacturing compact mechanical parts.

However, new emerging trends are gaining ground all over the world in the 3D-printed MEMS field, except for Italy, as for example the use of new AM techniques to fabricate MEMS devices. In the work of Blachowicz et al. [1], alternative fabrication technologies are shown to produce capacitive MEMS vibration sensors. For the MEMS cantilever, in particular, they presented 3D-printing technologies based on focused beams: a first one based on an ion beam that etched a silicon-on-insulator wafer, a second one based on an electron beam that selectively melted a Ti-alloy powder [1]. Another promising technology for MEMS electrothermal actuators is the laser-induced forward transfer, that allows one to deposit a metal layer at microscale with high accuracy using a laser and a reservoir made of a thin metal film deposited on a carrier substrate [131]. Then, other technologies that are spreading fast in the MEMS fabrication world are micro-transfer printing and sheet lamination. Micro-transfer printing is performed using thin layers of prepatterned material sheets that are stacked one on top of the other through a stamp [131]. Its working principle is close to sheet lamination, where sheets of material are stacked, cut, and bonded together using an energy source, like heat or ultrasonication, or with a glue [132].

The restriction of materials in each 3D-printing technique can be considered as a challenge; indeed, the opportunity to employ novel materials, distinct from the commonly used silicon, such as polymeric, ceramic, and metallic materials, has enabled the diverse application of MEMS across various scenarios. This approach leverages the unique characteristics of these materials, allowing for overcoming the limitations associated with the exclusive use of silicon.

Nevertheless, further efforts must be made to create solutions that can be compatible with multiple application fields, such as the biomedical, especially in order to enhance some important features like the biocompatibility of the materials, which should be considered as one limitation in the manufacturing of Bio-MEMS [133].

In addition, in the Italian scenario, fewer materials are exploited than in the rest of the world, like different piezoelectric materials [134]—such as PVDF, PEGDA, BaTiO_3_, or a combination of them, which can be employed for a variety of applications—as well as other polymers, like photopolymer resins or a composite materials, which show great potential in the production of MEMS [135].

Summarising, the fabrication of MEMS by additive manufacturing technologies is a cornerstone of next-generation innovation, and in this context, the know-how developed by the Italian community appears crucial both in industry and in academia. As a natural consequence, the necessity emerges to strengthen national and international networking, to bring to everyday life the discoveries here presented.

## Figures and Tables

**Table 1:** A summary of the mentioned AM technologies’ characteristics with their advantages and disadvantages, grouped following the ISO 52900:2021.

Group	Name	Characteristics	Advantages	Disadvantages	Ref.
Vat photopolyerisation technologies	SL	Laser-induced point-to-point photopolymerisation of a resin inside a tank, layer-by-layer growth of printed objects over a Z-moving building platform	High accuracy at microscale Good resolution (tens µm) High surface quality High complexity geometries allowed	High costs (because of laser) Slow printing process (depends on printing parameters) Need for post-curing process Need for support structures for overhangs	[5,14,15]
	DLP	Projector-induced layer-by-layer photopolymerisation of a resin inside a tank, growth of printed objects over a Z-moving building platform	Fast printing process (depending on the printing parameters) High accuracy at microscale Good resolution (tens µm) High surface quality High-complexity geometries allowed High-throughput process	Great issues of shrinkage Moderately expensive (compared to other commercially available technologies) Need for post-curing process Need for support structures for overhangs	[5,14]
	TPP	Laser-induced photopolymerisation inside a photoresist drop, growth of printed objects over a glass substrate (typically)	No need for a liquid-filled tank High resolution at submicrometric scale (depending on objective’s numerical aperture and material–light interaction) High-complexity geometries allowed No need for support structures Multi-material structures allowed Flexible and precise printing process	High costs (because of laser) High printing time (due to printing mechanisms) Hardly scalable fabrication technology Limited light intensity working window	[14,16,17]
Material extrusion	FFF	Extrusion of a semi-molten thermoplastic filament through a heated nozzle, layer-by-layer growth of printed objects over a heated building platform	Low costs Wide range of materials High stability of the printed parts No need for post-processing steps Fast printing process (depending on printing parameters) Scalability	Poor resolution (hundreds of µm) Bad surface quality (staircase effect can appear) Average accuracy Need for support structures for overhangs	[5,14,19,20]
Material jetting	MJM/ PolyJet	Drop-by-drop deposition of two photosensible materials, and consequent photopolymerisation through a lamp, on a Z-moving building platform. Layer-by-layer growing of objects together with support structures	High accuracy (at sub-millimetric scale) High resolution (at sub-millimetric scale) Good surface quality Clean process (thanks to closed printing chamber) Easy part detachment from platform	Proprietary materials Bad performance with high-aspect ratio parts	[12,14,21,22,23]
	IJP	No-contact direct writing of solutions or dispersions, drop-by-drop or continuous deposition through a thermal, piezoelectrical, or electrodynamical Z-moving head on flexible or rigid substrates, moving along XY plane	High resolution (depending on printing parameters and ink properties) High spatial accuracy Fast solvent evaporation (heating the substrates)	Final result strongly affected by substrate wetting properties Slow printing process (depending on printing parameters) Limited range of materials	[14,16]
	Pyro-EHD	Nozzle-free inkjet technology; deposition of liquid inks relies on a pyroelectric effect, thanks to a heating system that heats up an LN crystal and, consequently, the microscope glass ink reservoir. The drops are deposited on the substrate, positioned on a moving platform, and monitored with a camera.	High resolution High accuracy Deposition on already-existing 3D structures	Low reproducibility Need for continuous control of distance between substrate and reservoir	[14,16,24]
Powder bed fusion	µMLS	Metal submicron powders spread on a Z-moving heated platform from a powder reservoir are sintered by a 100 W ÷ 2 kW Nd:YAG laser with spot size 50 ÷ 180 nm. The process is performed in a closed chamber to avoid undesired chemical reactions, and the whole system is on an antivibration table.	High resolution (at micrometric scale) High accuracy High quality and smooth surfaces Prevention of powder agglomeration Assemblies and movable parts printed together No need for support	Post-processing required High costs (because of laser and metallic powders) Slow printing process Requires high quantity of metallic powders	[14,25,26,27]
Powder bed fusion	SLM	Powder reservoir filled with metal powders that are spread on a Z-moving printing bed, where a high-energy IR Yd:YAG laser selectively melts the material. The process is performed in a closed chamber filled with inert gases. The powder residues are eliminated through processes like EDM	Complete and dense near-net-shaped parts (if printed parameters are optimised) One-step fabrication No binders required No need for support Stacking of printed parts Reproducibility High-quality surface	Limited resolution (depending on particles’ dimension and layer thickness) Limited accuracy High costs (because of laser) Small particles agglomeration Layers adhesion problems (related to heat capacity, latent heat, and laser energy dose)	[14,16,28]

**Table 2:** A summary table to compare the current application of AM technologies for MEMS fabrication in Italy with the current application of them in the rest of the world.

AM Technology	Current Application (Italy)	Current Application (World)	Ref.
SL	Holder for a quartz crystal microbalance Coriolis mass flowmeter One-/Three-axes accelerometer Magnetic actuator Microfluidic devices VOCs monitoring systems Supercapacitors Cantilevers	Semicircular channel for flow sensor MEMS sensor for tensile strength Electrothermal micro-actuator Microfluidic devices	[1,10,29,30,31,32,33,34,35,36,37,38,43,131]
DLP	Microfluidic devices Micro-cantilever Photocontrallable Valve Nanogenerators Sensors Actuators	Piezoelectric microphone Microfluidic devices	[1,39,40,42,131]
TPP	Micro-optics Nano-resonator Microgrippers	Photonic crystal F.C.C. Clock Optically driven manipulator mechanism Light propagator Accelerometer	[1,17,44,45,131]
FFF	IMU Differential capacitive accelerometer Microfluidic devices	Pressure sensor Switch Magnetic actuator Antennas	[1,11,19,20,37,46,47,48,131]
MJM/PolyJet	Soft robotic actuator with integrated pressure sensor Scan head Microfluidic devices	Bio-inspired finger system Vibrational tactile actuator Holder for CCD camera Microfluidic devices	[1,2,8,12,49,50,131]
IJP/Pyro-EHD	Microlenses Probe Metal–insulator–metal capacitors	Strain gauge sensor Conductive thin electrodes	[1,24,51,131]
MLS	Flying probe ADM for TFEPAS Sensors Uniaxial capacitive accelerometer	Metal traces for microelectronics Battery packaging	[1,52,53,131]
SLM	Biaxial/capacitive accelerometer Micro-gear	Microgrippers	[1,54,55,131]

## Data Availability

No new data were created or analyzed in this study. Data sharing is not applicable to this article.
